# dMyc Functions Downstream of Yorkie to Promote the Supercompetitive Behavior of Hippo Pathway Mutant Cells

**DOI:** 10.1371/journal.pgen.1001140

**Published:** 2010-09-23

**Authors:** Marcello Ziosi, Luis Alberto Baena-López, Daniela Grifoni, Francesca Froldi, Andrea Pession, Flavio Garoia, Vincenzo Trotta, Paola Bellosta, Sandro Cavicchi, Annalisa Pession

**Affiliations:** 1 Dipartimento di Patologia Sperimentale, Alma Mater Studiorum, Bologna, Italy; 2 National Institute for Medical Research, London, United Kingdom; 3 Dipartimento di Biologia Evoluzionistica Sperimentale, Alma Mater Studiorum, Bologna, Italy; 4 Dipartimento di Ginecologia, Ostetricia e Pediatria, Alma Mater Studiorum, Bologna, Italy; 5 NGB Genetics s.r.l, University of Ferrara, Ferrara, Italy; 6 Department of Biology, City College of the City University of New York, New York, New York, United States of America; Stanford University, United States of America

## Abstract

Genetic analyses in *Drosophila* epithelia have suggested that the phenomenon of “cell competition” could participate in organ homeostasis. It has been speculated that competition between different cell populations within a growing organ might play a role as either tumor promoter or tumor suppressor, depending on the cellular context. The evolutionarily conserved Hippo (Hpo) signaling pathway regulates organ size and prevents hyperplastic disease from flies to humans by restricting the activity of the transcriptional cofactor Yorkie (*yki*). Recent data indicate also that mutations in several Hpo pathway members provide cells with a competitive advantage by unknown mechanisms. Here we provide insight into the mechanism by which the Hpo pathway is linked to cell competition, by identifying dMyc as a target gene of the Hpo pathway, transcriptionally upregulated by the activity of Yki with different binding partners. We show that the cell-autonomous upregulation of dMyc is required for the supercompetitive behavior of Yki-expressing cells and Hpo pathway mutant cells, whereas the relative levels of dMyc between Hpo pathway mutant cells and wild-type neighboring cells are critical for determining whether cell competition promotes a tumor-suppressing or tumor-inducing behavior. All together, these data provide a paradigmatic example of cooperation between tumor suppressor genes and oncogenes in tumorigenesis and suggest a dual role for cell competition during tumor progression depending on the output of the genetic interactions occurring between confronted cells.

## Author Summary

One of the major challenges of developmental biology and cancer research is to get a better understanding of how different signals regulate proper organ growth and prevent tumor formation. Even though there is a strong correlation between tumor progression and Myc family misexpression or Hippo signaling pathway malfunction, the relationship between these organ growth regulators remains unclear. Here, we demonstrate that the Hippo signaling pathway controls the transcription of *Drosophila dmyc*. Furthermore, we show that the misregulated expression of dMyc in Hippo mutant cells elicits their proliferative expansion at the expense of normal surrounding cells. These findings reveal a molecular mechanism of cooperation between oncogenes and tumor suppressor genes that favors both tumor progression and wild-type tissue elimination. Additionally, our findings indicate a dual role for cell competition during the tumour progression depending on the cellular context.

## Introduction

Growth regulation requires the fine tuning between the rate of cell death and cell proliferation in developing organs. Studies in *Drosophila* have revealed that somatic cells within a growing epithelium compete with one another for contribution to the adult organ and this phenomenon, known as “cell competition” [1], is possibly conserved among organisms, for a review [2]. Cell competition was discovered several decades ago comparing the clonal growth parameters of *Drosophila* wild type cells (+/+) and slow-dividing *Minute/+* cells [1]. From those analyses and recent data [3], it has been concluded that the contact between wild type and slow-growing cells, in genetic mosaics, favors the positive selection and clonal expansion of faster cells (winners) at the expense of slow-dividing ones (losers), although eventually the final number of cells in the organs is unaffected [3]. The biological function of cell competition remains unclear but it is thought to contribute to tissue homeostasis by coordinating the rate of cell proliferation and cell death [4], [5]. One of the best examples illustrating cell competition was obtained from the analysis of *Drosophila myc* [4], [5], opening to the speculation that this phenomenon might play a role in tumorigenesis [2], [6], however the basis of cell competition in tumorous situations has just begun to be investigated [7]. *dmyc* is an evolutionarily conserved proto-oncogene associated with different cellular processes, including cell cycle progression, cell growth and apoptosis [8]–[11]. The function of dMyc protein is both necessary and sufficient to control rRNA synthesis and ribosome biogenesis [12]. In *Drosophila*, cells carrying hypomorphic alleles of *dmyc* are viable in a homotypic context, but they are outcompeted and excluded from the epithelium when surrounded by wild type cells [5]. By contrast, *dmyc* overexpressing cells become “supercompetitors” able to kill wild type surrounding cells [4], [5]. Remarkably, dMyc upregulation is related with many types of human cancers [13] and it favors the clonal expansion of cells carrying additional oncogenic mutations [14], [15].

During the last years, the Hippo (Hpo) tumor suppressor pathway has emerged as a safeguard system restricting organ growth and preventing hyperplastic disease in metazoans [16], [17]. Mutations in several members of this pathway have been associated with tumor formation both in *Drosophila* and in humans [18]. It has also been reported that mutations in many members of the Hpo pathway can rescue the viability of heterozygous *M/+* cells in genetic mosaics [19], suggesting that these mutant cells behave as “supercompetitors”. Therefore the detailed analysis of Hpo pathway members appears to be an attractive model in which to evaluate the relationship between cell competition and tumor growth, as well as the molecular mechanisms required for this crosstalk. Hpo, Salvador (Sav) and Warts (Wts) constitute the core of the Hpo pathway that regulates by phosphorylation the downstream transcriptional co-activator Yorkie (Yki) [18], [20]. The hyperphosphorylated form of Yki is retained in the cytoplasm [21], [22], thereby preventing the expression of several target genes involved in cell proliferation control (*Cyclin E*, *E2F1*, *bantam* miRNA) [16], [23]–[25], cell death (*dIAP1*) [16] and cell signaling regulation (*dally* and *dally-like*) [26]. It has been demonstrated that Yki regulates its target genes by binding to Scalloped (Sd), a TEAD/TEF family transcription factor [27]–[30]. In addition, recent data indicate that Yki is also able to bind to the homeoprotein Homothorax (Hth) forming a complex which regulates the transcription of *bantam* in the eye disc [31]. The atypical cadherins Fat (Ft) [26], [32]–[37] and Dachsous (Ds) [20], [26], [33], [38], as well as the FERM-domain proteins Expanded (Ex) and Merlin (Mer) [39], have also been implicated in the pathway as upstream components. Although their biochemical functions are still uncertain, it is assumed that they converge on Wts to regulate Yki activity [40], [41].

Here we provide a detailed analysis of the autonomous and non-autonomous effects on growth of *yki*-expressing cells and mutations of members of the Hpo pathway. In addition we show that *dmyc* is a transcriptional target of Yki, able to confer competitive properties to the Hpo pathway mutant cells in the *Drosophila* wing. Furthermore, *dmyc* upregulation is essential to sustain the high rate of cell proliferation of Hpo mutant cells and to protect them from being eliminated in a competitive background. Finally, we show that the relative levels of dMyc protein between neighboring cells are critical in order to define the role of cell competition during tumor progression.

## Results

### Hpo pathway mutant cells display supercompetitive properties

**Figure 1:**
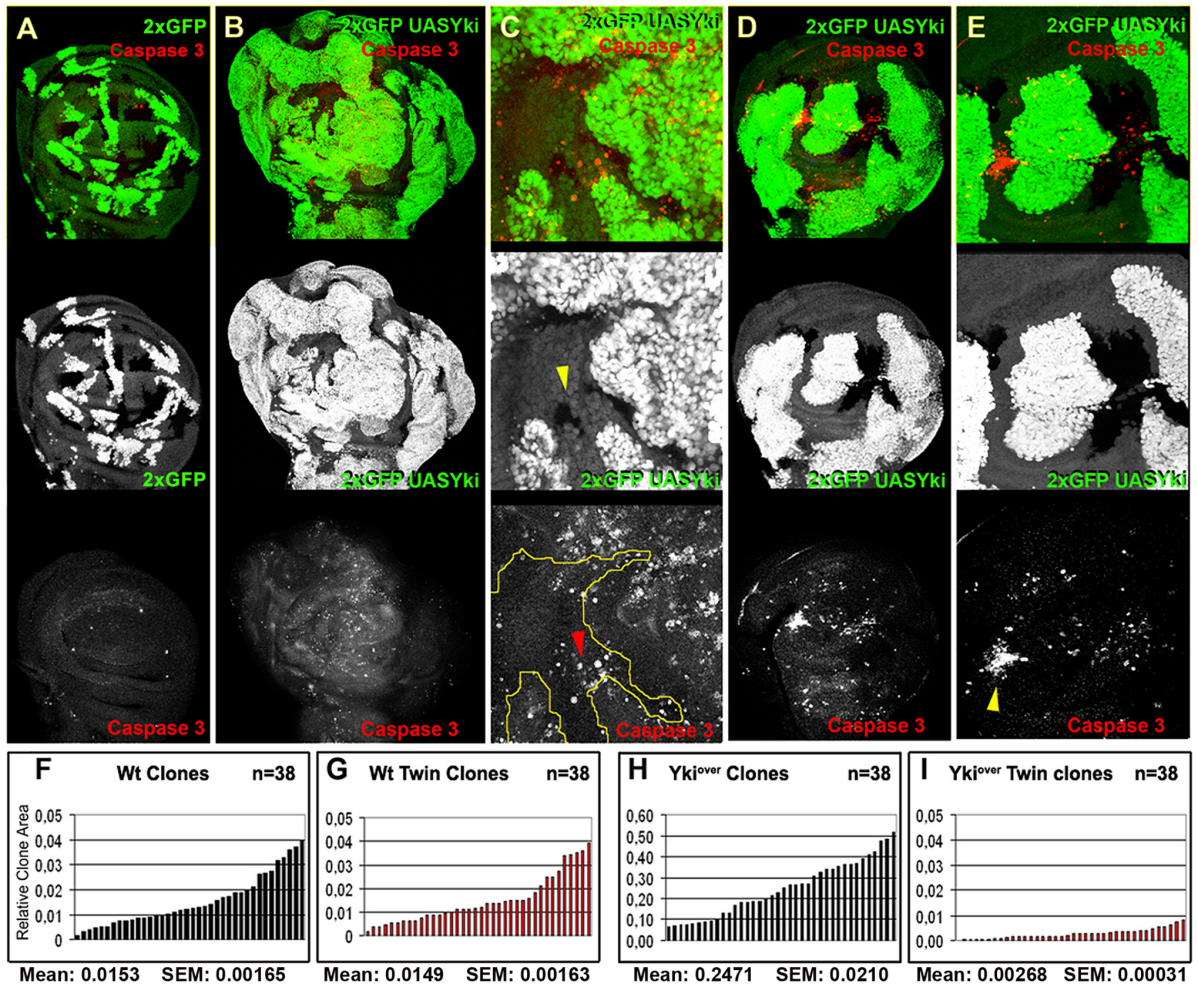
*yki* overexpression confers cells a supercompetitive behavior. (A) *yw*, hs-*Flp*, *tub-Gal4*, UAS-*GFP*; FRT42D, *tub-Gal80*/FRT42D, Ubi-*GFP* clones induced at 48–72 h AEL. Twin clones are marked by the lack of GFP (0xGFP); imaginal discs were dissected at 120h AEL. (B–E) *yw*, hs-*Flp,tub-Gal4*, UAS-*GFP*; FRT42D, *tub-Gal80*/FRT42D, Ubi-*GFP*; UAS-*yki*/+ clones (2xGFP = 2XUbi-GFP+tub-GFP) sorrounded by wild type cells. (B,D) Clones were induced at 24–48 h AEL and dissected at 120 h AEL (B) or 96h AEL (D). C and E are magnifications of B and D respectively. Cell death assayed by Caspase 3 staining is shown in red (A–E). Yellow arrowhead indicates a wild type twin in C (0xGFP), whereas the red arrowhead indicates wild type cells dying far from *yki* ^over^ clones. Note that wild type cells preferentially die when enclosed by *yki* ^over^ cells (yellow arrowhead in E). (F–I) Histograms showing the surface area of wild type and *yki* ^over^ clones and respective twins induced at 48–72 h AEL and allowed to grow until 120h AEL. (F, G) Wild type clones (F) and their twins (G) display the same size profile. (H) The size profile of *yki* ^over^ clones indicates that they are larger either than wild type controls (F, G) or than their wild type twins (I). Note also that wild type twins (I) of *yki* ^over^ clones (H) are smaller than wild type clones induced at the same stage of development (F, G). SEM = Standard Error of the Mean. *P*<0.0001.

In order to analyze the competitive properties of Hpo pathway mutant cells, we used mosaic analysis to compare the size of *yki* overexpressing clones (hereafter referred to as *yki* ^over^) with their wild type twins. While clones and twins showed a comparable size in the wild type control (Figure 1A, 1F, and 1G, and Figure S1A, S1C, S1D), *yki* ^over^ clones were notably larger than their wild type twins in wing discs dissected either 60h (Figure 1D, 1H, and 1I) or 48h (Figure S1B, S1E, S1F) after heat-shock induction. Furthermore, *yki* ^over^ wild type twins were almost disappeared from the epithelium at 120h after egg laying (AEL) (Figure 1B and 1C). These differences in size were also prominent when discs were dissected at 96h AEL (Figure 1D and 1E). Interestingly, the clonal expansion of *yki* ^over^ cells was also correlated with non-autonomous apoptosis, as revealed by active Caspase 3 immunoreactivity of a subset of surrounding wild type cells (Figure 1B–1E). The size advantage of *yki* ^over^ clones and the induction of apoptosis in wild type cells is consistent with the broadly assumed definition of cell competition, which implies that the clonal expansion of the winner cells occurs at the expense of the juxtaposed losers, that are eliminated by apoptotic death [2], [42], [43]. The pattern of cell death in wild type and *yki* ^over^ cells (Figure 1B–1E) was not confined to the interface between the two cell types; as can be seen in Figure 1E, cell death extends several cell diameters away and wild type cells tend to die massively when enclosed between nearby mutant clones (Figure 1E, yellow arrowhead). A similar pattern of non-autonomous cell death was observed in wild type cells nearby mutant clones for other members of the Hpo pathway, such as *ft* and *ex* (Figure S2A, S2B, S2C). Strikingly, *yki* ^over^ clones and *wts* mutant clones grown for a longer period presented autonomous cell death (Figure 1C, see active Caspase 3 staining, and Figure S2D), despite the upregulation of anti-apoptotic molecules such as dIAP1 [16]; this might be possibly due to either developmental constraints compensating for excessive proliferation of the entire organ or toxicity caused by high and constant levels of Yki. Altogether, these results confirm the previously suggested supercompetitive properties of the Hpo pathway mutant clones [19] by revealing their ability to overgrow and eliminate surrounding wild type cells.

### *dmyc* is a Hpo pathway target gene regulated by the activity of Yki

**Figure 2:**
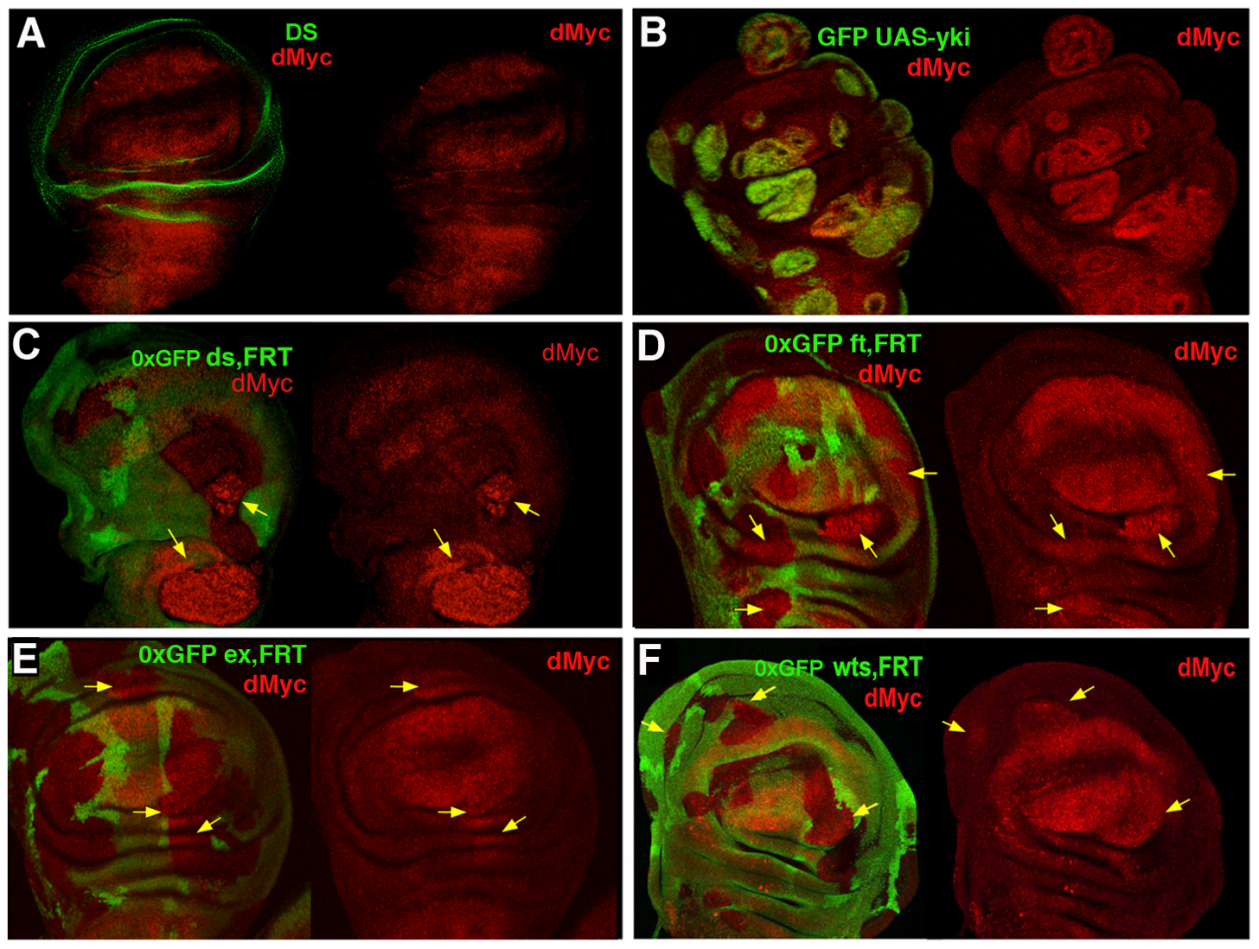
*dmyc* oncogene is regulated by the Hpo pathway. (A) Double staining with anti-dMyc (red) and anti-Ds (green) reveals that dMyc and Ds proteins show a complementary pattern of expression in the wing disc; dMyc is highly expressed in the wing pouch and less in the notum, while Ds localizes mainly in the hinge and pleura, where dMyc expression is the lowest. (B) dMyc staining of *yw*, hs-*Flp*; *actFRTy* ^+^ *FRTGal4*, UAS-*GFP*/UAS-*yki* Flp-Out clones (GFP^+^) show increased dMyc protein level. (C–F) dMyc protein levels are increased in *ds* ^d36^, *ft* ^G-rv^, *ex* ^E1^ and *wts* ^X1^ LOF clones (arrows). Clones were induced by the Flp/FRT system and are marked by the lack of GFP (0xGFP). Clones were induced at 42–54 h AEL (C–F) or at 54–66 h AEL (B).

It is well documented that the confrontation of different levels of dMyc protein between two populations of cells either *in vivo* [4], [5] or in cell culture [44] can trigger cell competition, however the molecular mechanism by which this occurs is unknown. In addition, *myc* family oncogenes are frequently overexpressed in human cancers and it contributes to tumor progression of *YAP*-expressing cells (mammalian orthologue of *yki*) [17]. We have previously shown that a transcriptional activation of *dmyc* occurs in *ft* mutant tissues and that *ft* clones fail to grow in a *dmyc* hypomorphic background [45], indicating a possible regulation of this oncogene by the Hpo pathway. Moreover, the expression pattern of dMyc is complementary to that of Ds in the wing imaginal disc (Figure 2A), suggesting a possible functional interaction. To validate this hypothesis, we analyzed dMyc expression in mutant clones for several members of the Hpo pathway and in *yki* ^over^ cells by immunofluorescence. Noticeably, we found that dMyc was upregulated in a cell-autonomous manner in *yki* ^over^ clones throughout the wing disc (Figure 2B and Figure S3), with the weakest activation in the lateral regions, and in a subset of clones mutant for several Hpo pathway members (Figure 2C–2F). These differences in dMyc activation between *yki* ^over^ clones and clones mutant for other members of the Hpo signaling pathway might be due to additional levels of regulation of the Hpo cascade operating on upstream members. According to our previous observations, we would predict a repression of dMyc upon Hpo pathway hyperactivation. To investigate this hypothesis, we expressed Hpo in the *spalt* expression domain of the developing wing disc. Since Hpo overexpressing cells die massively by apoptosis during development [25], we coexpressed the anti-apoptotic factor p35. As expected, cells coexpressing Hpo and p35 show reduced levels of dMyc with respect to the control (Figure S4A) in both late (Figure S4B) and early (Figure S4C) wing discs. Thus dMyc levels can be regulated by the Hpo pathway activity.

### *dmyc* is transcriptionally regulated by Yki

**Figure 3:**
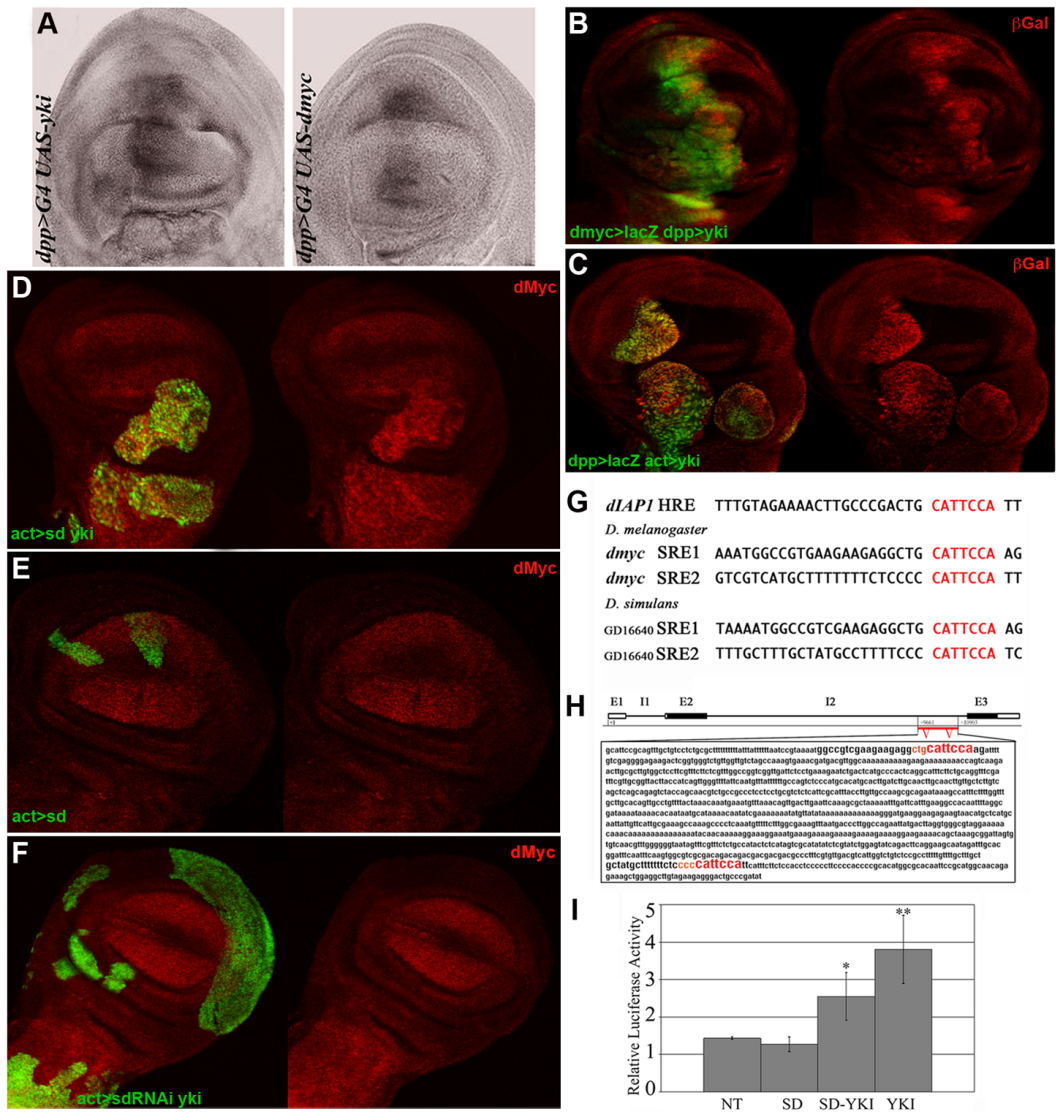
Yki regulates *dmyc* transcription. (A) *In situ* hybridization on imaginal wing discs with a full-length *dmyc* RNA probe. The *dmyc* transcript is robustly upregulated in the *dpp>yki* disc on the left. A *dpp>dmyc* disc is shown on the right as a control. (B) ßGal staining (red) of *dmyc>lacZ* ^G0354^/+; *dpp*>Gal4, UAS-*GFP*/UAS-*yki* imaginal wing discs. *dmyc* expression is upregulated in the *dpp* domain (GFP^+^). (C) ßGal staining (red) of *dmyc>lacZ* ^G0354^/hs-*Flp*; *act*FRT*y* ^+^FRT*Gal4*, UAS-*GFP*/UAS-*yki* imaginal wing discs. *yki* ^over^ clones (GFP^+^) were induced at 42–54h AEL. As can be observed, a robust activation of *dmyc* regulatory sequences is visible within the mutant clones. (D,E) dMyc staining of *yw*, hs-*Flp/+*; UAS-*sd*/+; *act*FRT*y* ^+^FRT*Gal4*, UAS-*GFP*/UAS-*yki* and *yw*, hs-*Flp/+*; UAS-*sd*/+; *act*FRT*y* ^+^FRT*Gal4*, UAS-*GFP*/+ imaginal wing discs respectively. Clones (GFP^+^) were induced at 42–54 h AEL. As can be observed in E, dMyc levels are comparable inside and outside the mutant clones. (F) dMyc staining of *yw*, hs-*Flp/+*; UAS-*sd*-RNAi/+; *act*FRT*y* ^+^FRT*Gal4*, UAS-*GFP*/UAS-*yki* imaginal wing discs. Clones (GFP^+^) were induced at 42–54 h AEL. No clones in the wing pouch region were recovered and those outside the wing pouch never took the tumorous shape typical of *yki* ^over^ clones. All larvae were dissected at 120h AEL. (G) *dIAP1* Hpo Responsive Elements (HRE) for Yki/Sd complexes are indicated in red [28]. The same consensus sites are found twice in the second intron of *dmyc* both in *D. melanogaster* and *D. simulans*, indicating a possible functional conservation. (H) Scheme of the *dmyc locus* showing the exons (E) and introns (I). The red bar indicates the region of the second intron containing the putative Yki/Sd consensus sites. (I) Luciferase assay on S2 *Drosophila* cells. NT: cells transfected with *dmyc-firefly* alone. *P* value is significative for Sd/Yki (*P*<0,05) and Yki (*P*<0,01). Error bars represent standard deviation (triplicate wells).

*dmyc* was observed upregulated in RT-PCRs performed on *ft* mutant imaginal discs [45], suggesting that it could be a transcriptional target of the Hpo pathway. In order to investigate this, we first performed an *in situ* hybridization in *Drosophila* wing discs expressing *yki* under the control of the *decapentaplegic* (*dpp*) promoter. As expected, *dmyc* transcript is detectable in the *dpp* domain both in *yki* and control *dmyc*-expressing discs (Figure 3A). No signal within the *dpp* domain was detected in *dpp>GFP* control discs (not shown). We were able to reproduce these data using a *dmyc>lacZ* line [46] which recapitulates accurately the *dmyc* pattern throughout the wing disc during development [7], [47]. As can be seen in Figure 3B, the ßGal expression is increased in the *dpp* domain upon *yki* expression, indicating that Yki acts upon *dmyc* transcription. This result was supported using clonal analysis, both in *yki* ^over^ cells, as shown in Figure 3C, and in cells mutant for *ft* (Figure S5). Altogether, these data demonstrate the ability of the Hpo pathway to regulate *dmyc* transcription in the imaginal wing disc.

Yki transcriptional activity depends on the formation of tissue-specific complexes with different partners such as Scalloped and Homothorax [27]–[31]. In order to study the contribution of Sd to *dmyc* upregulation by Yki in the wing disc, we generated *yki* ^over^ clones coexpressing either a UAS-*sd* or a UAS-*sd*-RNAi construct (see Figure S6A for validation). As can be seen in Figure 3D, *sd* ^over^; *yki* ^over^ clones overgrew relative to *yki* ^over^ clones (compare with Figure 2B, 68% increase on average, n = 27, *P*<0,005) confirming previous data [29], but we were not able to detect significant differences in dMyc protein levels compared to *yki* ^over^ clones (n = 22, *P* = 0,43). As expected, control *sd* ^over^ clones did not overgrow and did not deregulate dMyc (Figure 3E), demonstrating that Yki is required for dMyc upregulation. We were not able to recover *sd*-RNAi; *yki* ^over^ clones in the wing pouch region, but clones generated in other territories of the wing disc, although large, did not upregulate dMyc (Figure 3F), nor showed the same degree of hyperplasia as Yki expression alone (Figure 1B–1E). *sd*-RNAi control clones were very small and did not deregulate dMyc (not shown). These data indicate a key role for Sd *in vivo* in upregulating dMyc in *yki* ^over^ clones, and in contributing to the *yki* ^over^ tumorous phenotype.

Interestingly, examination of *dmyc locus* revealed the existence of several CATTCCA repeats in non-coding regions of the gene, which perfectly match the mammalian [48], [49] and *Drosophila* [28], [29] TEAD/TEF family transcription factor consensus binding motifs (mammaliam orthologues of Scalloped). In addition, these putative binding motifs for Yki/Sd complexes are evolutionarily conserved in *D. simulans* (Figure 3G) and relatively close to the insertion point of P elements that recapitulate the endogenous expression of the gene (*dm* ^PL35^ LacZ [50], [51] and *dm* ^BG02383^ Gal4 insertions - http://flybase.org/reports/FBti0018138.html).

To test the significance of these sequences in *dmyc* regulation, we generated a *dmyc*-*firefly* reporter containing the putative responsive elements for Yki/Sd complexes (Figure 3H) and performed a transient dual luciferase assay in S2 cells. As can be seen in Figure 3I, the reporter was specifically activated upon Sd and Yki cotransfection but, unexpectedly, the transfection of Yki alone was able to activate the reporter as efficiently as the cotransfection Yki/Sd (Figure 3I). This result suggests that in presence of high levels of Yki alone, additional partners such as Hth [31] could bind it and co-regulate *dmyc* expression.

Indeed, complementarily to Yki/Sd complexes, Yki/Hth complexes seemed to play the same role in the presumptive thoracic region of the wing disc. Supporting this conclusion, *hth*-RNAi; *yki* ^over^ clones down-regulated dMyc in the notum (30% reduction on average, n = 15, *P*<0,05, Figure S6B, yellow arrows) and did not grow as tumors in that region. By contrast, they were undistinguishable from *yki* ^over^ clones in the wing pouch region (Figure S6B, white arrowhead), where Hth expression is almost undetectable (Figure S6C). Altogether, these latter results indicate that Sd and Hth play a role in Yki-induced tumorigenesis by regulating *dmyc* expression in the wing disc, with Sd playing a more critical role in the pouch and Hth acting in the presumptive thorax.

### dMyc upregulation enhances cell proliferation of the Hpo pathway mutant cells in an autonomous manner

**Figure 4:**
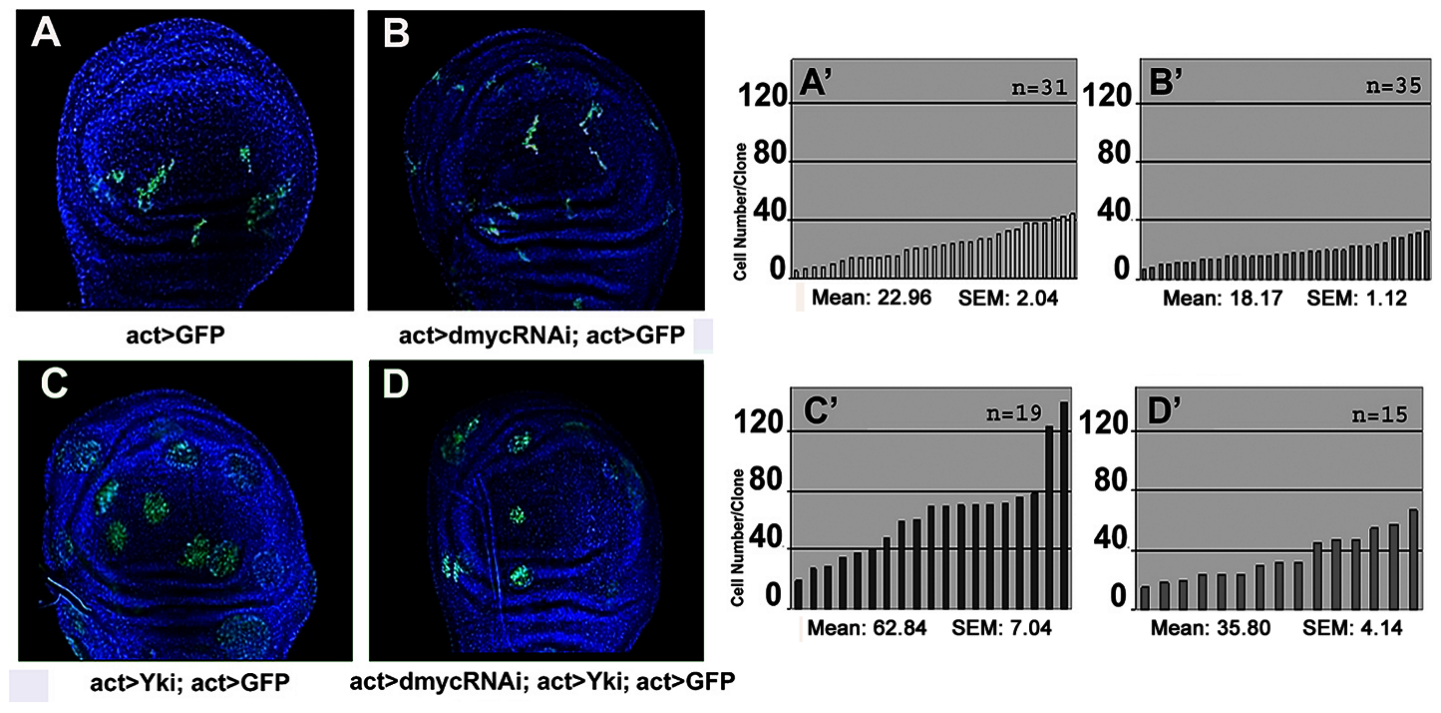
dMyc boosts the proliferative abilities of *yki* ^over^ cells. Four types of clones were simultaneously induced through the Flp-Out system at 48–72h AEL and allowed to grow for a comparable period (until 120h AEL). Clones are GFP^+^, nuclei are counterstained with DAPI. (A–A′) *act*>GFP control clones. (B–B′) *act*>*dmyc*-RNAi clones. (C–C′) *act*>*yki* ^over^ clones proliferate faster than wild type clones. (D–D′) *act*>*dmyc*-RNAi; *yki* ^over^ clones: proliferation is reduced relative to *act*>*yki* ^over^ clones. (A′–D′) Histograms showing the number of cells/clone of the genotypes indicated in A–D. SEM = Standard Error of the Mean. *P*<0.01.

With the aim to investigate the cell-autonomous contribution of dMyc overexpression to *yki* ^over^ phenotypes, we first compared the size of *yki* ^over^ clones with that of *yki* ^over^; *dmyc*-RNAi clones (Figure 4, see also Figure S7A, S7A′, and [7] for RNAi construct validation ). As expected, *dmyc*-RNAi clones showed a reduced number of cells with respect to that observed in wild type clones (21% reduction on average, compare Figure 4B and 4B′ with Figure 4A and 4A′, *P*<0.01). The reduction in cell number displayed by the *yki* ^over^; *dmyc*-RNAi clones with respect to the *yki* ^over^ clones was even more evident (43% reduction on average, compare Figure 4D and 4D′ with Figure 4C and 4C′, *P*<0.01), and this percentage raised up to 65% (n = 87, *P*<0,001) when these clones were induced earlier in development (42–54h AEL), indicating a strong cell-autonomous requirement of dMyc protein for the expansion of *yki* ^over^ clones. We also observed that the non-autonomous apoptosis induced by *yki* overexpression was reduced upon *dmyc* deprivation (32% on average, n = 28, *P*<0,01, Figure S7B). These data suggest that dMyc upregulation promotes cell proliferation of *yki* ^over^ clones in an autonomous manner, and also promotes their competitive behavior.

**Figure 5:**
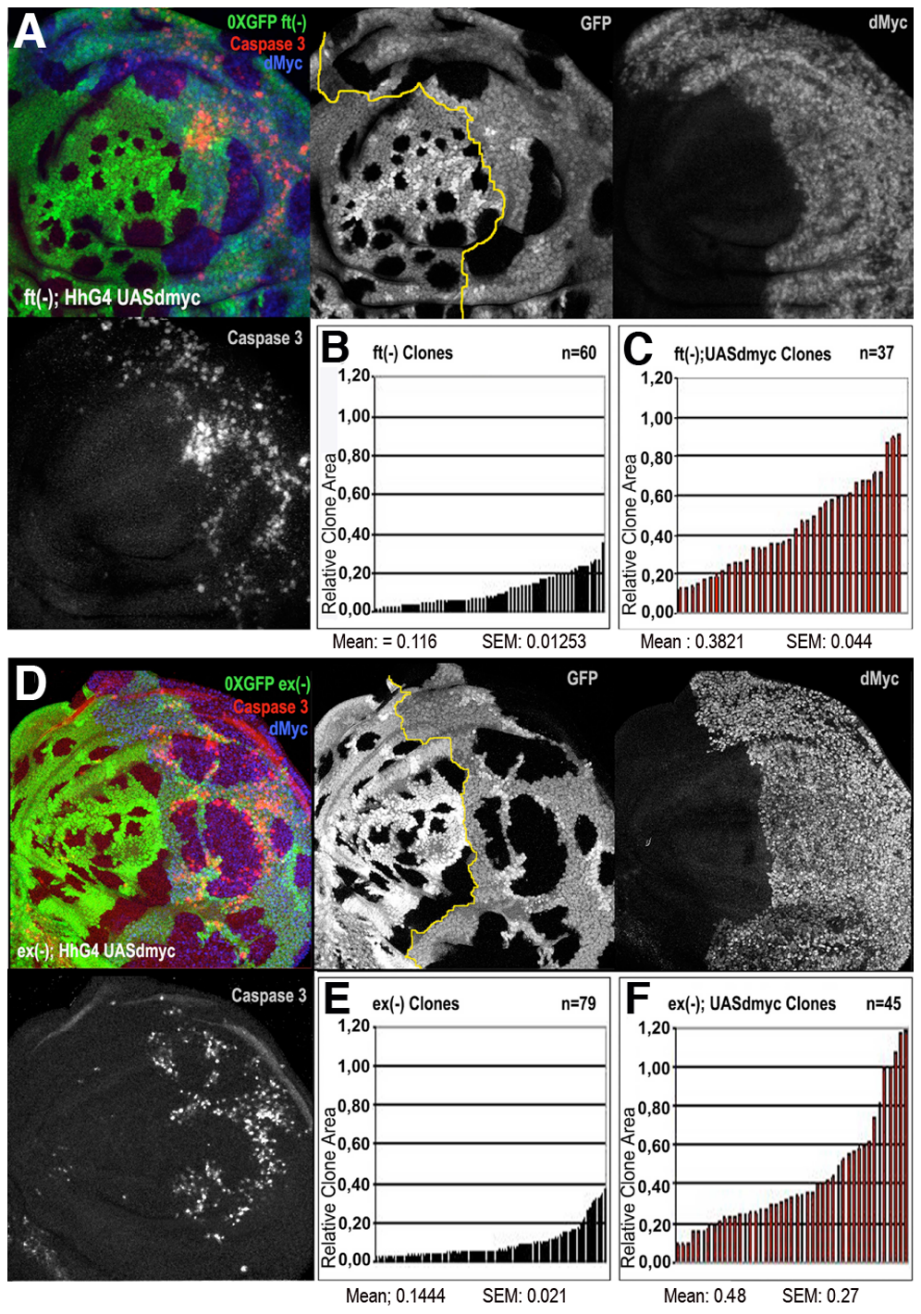
dMyc overexpression enhances the proliferation of Hpo pathway mutant cells. *ft* (A–C) and *ex* (D–F) LOF clones (0xGFP) generated in a background where posterior (P) cells ectopically express *dmyc* under the control of *hh-Gal4* (A and P compartments are separated by a yellow line in A and D; P is on the right). dMyc overexpression strongly enhances the proliferative activity of *ft* (A–C) and *ex* (D–F) mutant cells; mutant clones are larger in dMyc-expressing territories (P compartment in histograms C and F) than in a wild type background (A compartment in histograms B and E). SEM = Standard Error of the Mean. *P*<0.001. High levels of active Caspase 3 signal are evident in dMyc-expressing cells outside *ft* and *ex* mutant clones in the posterior compartment.

To further characterize this proliferation-promoting effect of dMyc, we compared the clonal behavior of various mutations in members of the Hpo pathway grown in two different genetic backgrounds: a wild type context and a genetic background overexpressing *dmyc* under the control of a *hedgehog* promoter in the posterior (P) compartment of the wing disc. We found that *ft*, *ex* and *ds* mutant clones were consistently larger in those territories expressing uniform levels of dMyc than in the wild-type background (Figure 5 and Figure S8). It is however described that the overexpression of dMyc is able to autonomously increase apoptosis [8]–[11]. In fact, the wild type tissue expressing high amounts of dMyc tends to die and does not overgrow (see active Caspase 3 stainings in Figure 5A and 5D). Noticeably, the apoptosis mediated by dMyc overexpression seems to be extremely reduced inside *ft* and *ex* clones (Figure 5A and 5D) with respect to the wild type surrounding territories, likely due to the upregulation of antiapoptotic genes such as *dIAP1*, a target of the Hpo pthway [20]. In addition, the dying cells in this genetic background might induce morphogens to promote compensatory proliferation [52] that may contribute to the extra-growth of *ft*- or *ex*-UAS-*dmyc* expressing clones. To circumvent this problem, we repeated the same experiment coexpressing *dmyc* and *dIAP1*. As can be seen in Figure S9, both *ft* (Figure S9A) and *ex* (Figure S9D) mutant clones grown in the P compartment were still consistently larger than those originated in the A compartment, thus confirming a specific cooperation of *dmyc* and Hpo pathway mutants in clonal expansion.

Hpo mutant cells therefore seem to show the ability to take advantage of the cell mass accumulation boosted by dMyc overexpression to proliferate faster.

### dMyc upregulation prevents the Hpo pathway mutant clones from being restrained in a competitive background

**Figure 6:**
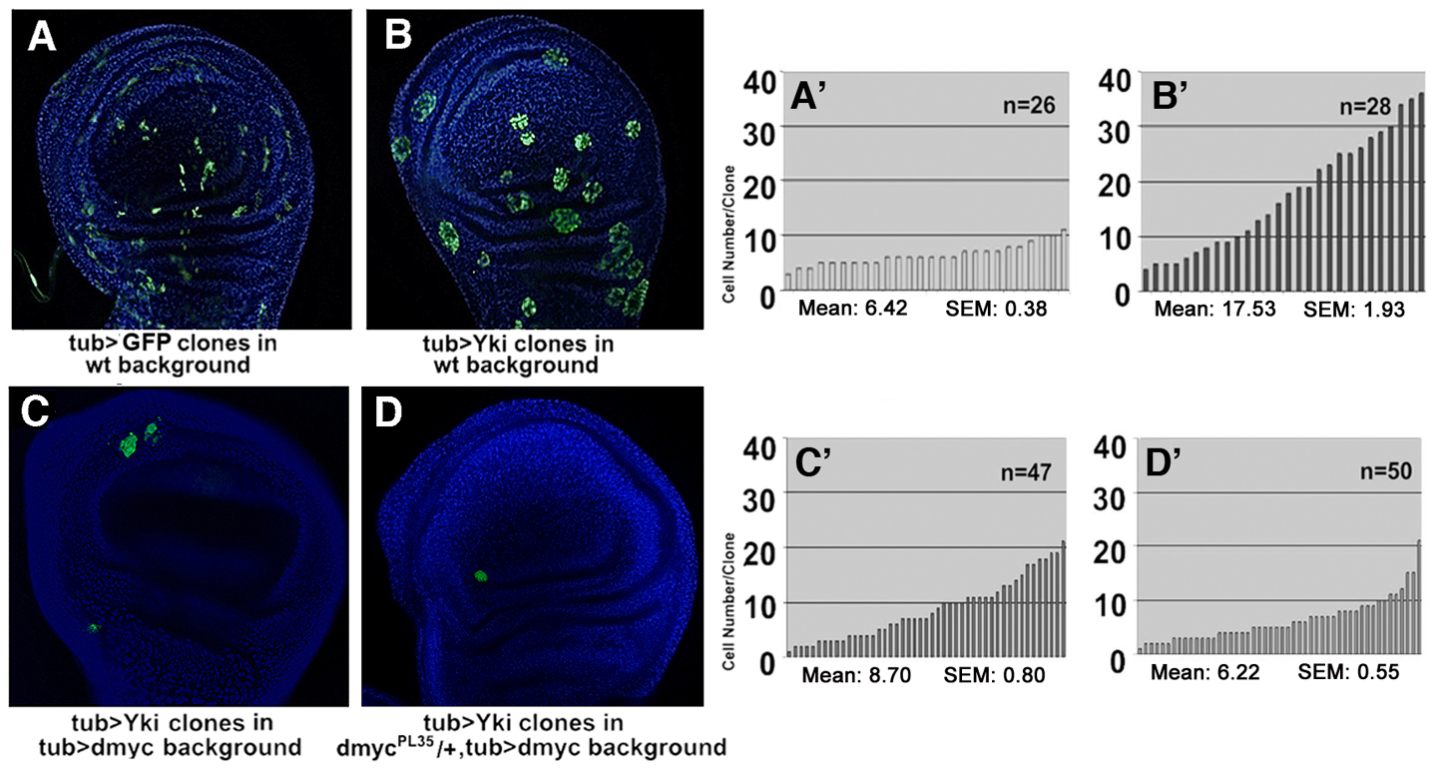
*yki^over^* clonal expansion is restrained by *dmyc*-induced cell competition. (A–D′) Cell competition assay shows that, while wild type clones are outcompeted in this genetic background [5], the clonal expansion of *yki* ^over^ cells is partially restrained. Clones were induced at 60–84 h AEL and allowed to grow until 120h AEL. Clones are GFP^+^, nuclei are counterstained with DAPI. (A–A′) wild type control clones generated through a *tubulin* (*tub*) Flp-OUT system. (B–B′) *yki* ^over^ clones generated with the same system as the wild type control. (C–C′) *yki* ^over^ clones in a *tub*>*dmyc* background are smaller than in a wild type background. *P*<0.01. (D–D′) *dmyc^PL35^/+*; *tub>yki* ^over^ clones in a *tub>dmyc* background are smaller than in C (*P*<0.01), confirming that the relative dMyc levels outside *vs* inside the clones affect the competitive ability of *yki* ^over^ cells. (A′–D′) Histograms showing the number of cells/clone of the genotypes indicated in A–D. In C and D discs are larger due to the overall increase in body size of *tub>dmyc* individuals [59] SEM = Standard Error of the Mean.

To address the non-autonomous relevance of *dmyc* upregulation in providing *yki* ^over^ cells with a supercompetitive behavior, we compared the size of *yki* ^over^ clones generated in a wild type background to that of *yki* ^over^ clones generated in a background ubiquitously overexpressing *dmyc* under the control of a *tubulin* (*tub*) promoter (*cell competition assay*, [4], [5]). In this assay cells express the endogenous *dmyc* gene plus an extra copy of the gene under the control of a *tub* promoter that ensures two-to-threefold increase of *dmyc* transcript [5]. This extra copy of *dmyc* is located in a removable cassette between the *tub* promoter and a Gal4 cDNA. Upon *dmyc* cassette excision, the *tub* promoter drives Gal4 expression in the clones and, as a result, those cells express lower levels of *dmyc* relative to the background and are rapidly eliminated from the tissue by cell competition. Only few genes have so far been found whose overexpression rescues cell viability in this context [5]. The relative difference in dMyc levels between *yki*-expressing cells and the surrounding *tub>dmyc* cells was minimized in a competitive background compared to a wild type context (compare Figure S7C and S7C′ with Figure 2B). In this competitive background, *yki* ^over^ clones showed a diminished ability to overgrow compared to a wild type background (44% reduction on average, compare Figure 6C and 6C′ and Figure 6B and 6B′; *P*<0,01). Besides the reduction in size, *yki* ^over^ clones showed an important reduction in clone number both in discs (Figure 6C) and adult wings (compare Figure S7E to Figure S7D). Moreover, *yki* ^over^ clones induced earlier in development (42–54h AEL) were never recovered at the end of larval development (not shown). These data indicate that the competitive properties of *yki* ^over^ cells are extremely reduced when they are surrounded by cells expressing very high amounts of dMyc.

We then performed the same competition assay as before while reducing *dmyc* activity inside the clones. We used the pupal lethal *dmyc* ^PL35^ allele [49] and, taking advantage of *dmyc locus* association to chromosome X, we were able to analyze both female (heterozygous condition, the expression of *dmyc* is halved) and male (hemizygous condition, the expression of *dmyc* is completely removed) larvae. In *dmyc* ^PL35^/+; *tub>dmyc* females, *yki* ^over^ clones were smaller than those described in the previous assay (28% reduction on average, compare Figure 6D and 6D′ to Figure 6C and 6C′, *P*<0,05), whereas they were completely outcompeted by 48h after the heat shock in males (not shown). Since it has been observed that a *dmyc* ^PL35^ heterozygous condition does not impair cell growth or proliferation rate [49], our results reveal an important role for *dmyc*-induced cell competition in controlling the clonal expansion of *yki* ^over^ cells, which may occur via their non-autonomous capabilities to compete with neighboring wild type cells.

### dMyc expression alone is not sufficient to prevent the elimination of *yki* mutant cells

*yki* LOF clones generated in a wild type background are not able to grow [16], [25] and the ectopic expression of the antiapoptotic proteins dIAP1 [25] or p35 (Figure S10A) poorly rescues their viability, whereas a *Minute* background [53] or *bantam* overexpression within *yki* clones has been shown to partially rescue their growth [25]. Since our results have indicated that *dmyc* participates in tumor growth of the Hpo pathway mutant cells, we therefore analyzed if the expression of dMyc was sufficient to prevent the death of *yki* mutant cells. The overexpression of dMyc failed to rescue the viability of *yki* ^−/−^ cells (Figure S10B). Since *yki* mutant cells express low levels of the apoptosis inhibitor dIAP1 (not shown), this result is not surprising, considering the autonomous cell death described for cells overexpressing dMyc [11]. However, *yki* mutant cells coexpressing dMyc and p35 also failed to grow (Figure S10C). The lack of expression of additional antiapoptotic genes and cell cycle regulators [18] possibly impedes the clonal growth of *yki* mutant cells even though they overexpress dMyc. This result suggests that *dmyc* expression is able to enhance the ability of Hpo pathway mutant cells to grow, but it is not sufficient to rescue tissue growth of *yki* ^−/−^ clones.

## Discussion

Cells within a tissue coordinate and execute complex genetic programs in order to succeed in completing a variety of processes during development. In this context, the phenomenon of cell competition may be part of the developmental plan that ensures removal and replacement of defective cells in growing organs, thus keeping their size invariant. In this work, we have evaluated in details the relationships between the phenomenon of cell competition and the clonal expansion of tumorous cells, using for that purpose mutants in components of the evolutionarily conserved Hpo pathway. From our studies we reveal that the Hpo pathway regulates dMyc expression, and show that this is critical for the tissue growth and competitive behavior of Hpo pathway mutant clones.

### dMyc is a Hpo pathway transcriptional target

*dmyc* upregulation has been demonstrated in many studies to provide cells with supercompetitive properties [4], [5], [7]. The model explaining how dMyc can confer competitive properties to cells is based on the relative levels of this protein in neighboring cell populations, transforming those cells expressing higher levels of dMyc into supercompetitors [4], [5]. *dmyc* overexpression is nevertheless insufficient to drive tumorous growth; *dmyc* ^over^ clones fail to overproliferate and show strong autonomous apoptosis [9]. Interestingly, we found that dMyc protein is overexpressed in Hpo pathway mutant clones, indicating an involvement for this cascade in *dmyc* regulation (Figure 2). Furthermore, the upregulation of dMyc in Yki-expressing cells correlates with an increase in the amount of mRNA, observed by *in situ* hybridization (Figure 3A) and using a *dmyc>lacZ* line (Figure 3B and 3C). Finally, we have identified a regulatory region in the second intron of *dmyc* that is sensitive to Yki abundance; importantly, this regulatory region includes predicted consensus-binding motifs for Sd (Figure 3H). Clonal experiments in the wing disc indicate that Sd is necessary for Yki function *in vivo*, since upon Sd downregulation Yki is no longer able to induce tumorous growth and does not upregulate dMyc (Figure 3F). All these findings support the notion that there is a transcriptional regulation of dMyc mediated by Yki/Sd complexes in the wing pouch. Importantly, similar results were observed for dMyc regulation in the notum by Yki/Hth complexes, suggesting that tumor growth and *dmyc* regulation are tissue-specific.

### What is the contribution of dMyc to the Hpo pathway mutant phenotypes?

We found that dMyc upregulation is a common feature of Hpo pathway mutant cells. Since *dmyc* has been repeatedly associated with tumor progression and cell competition, we analyzed its role in the clonal expansion of Hpo pathway mutant cells. We observed that the reduction of dMyc expression restricts the ability of Hpo pathway mutant cells to proliferate (Figure 4), whereas its uniform overexpression strongly promotes their proliferation (Figure 5). Furhermore, while dMyc-expressing wild type cells surrounding mutant clones are rapidly eliminated by autonomous apoptosis, Hpo pathway mutant cells are able to take advantage of dMyc role in protein biosynthesis and cellular growth to divide rapidly. This is a clear example of functional cooperation between different genes in order to favor tumor progression, but it also indicates a specific role of dMyc in promoting the clonal expansion of Hpo pathway mutant cells. According to these data, we conclude that dMyc behaves as a growth-promoting factor which sustains the hyperplastic phenotype of Hpo pathway mutant cells. Importantly, this specific cooperation might be evolutionarily conserved, since *c-myc* appears to be upregulated in a murine model of YAP-induced carcinoma [17].

### Relative levels of dMyc in neighboring cells restrict/promote clonal expansion of hyperplastic cells, likely through cell competition

It has been suggested that cell competition may be a mechanism potentially restricting the clonal expansion of tumorous cells [7], but it might also help faster proliferation of transformed cells. Our data indicate that Hpo pathway mutant cells are able to use high levels of dMyc to proliferate rapidly (Figure 5), but in a competitive context, where neighboring cells express high levels of dMyc, clonal expansion of *yki* ^over^ cells is restrained (Figure 6), therefore suggesting a tumor suppressor role for cell competition. Conversely, dMyc upregulation in *yki* ^over^ clones grown in a wild type background favors their clonal expansion promoting cell autonomous proliferation and also conferring the ability to outcompete sourrounding cells in a non-autonomous manner. These findings suggest that the phenomenon of cell competition may play a dual role in tumor progression depending on the output of the genetic interactions occurring between adjacent cells.

In summary, we have shown a tumor-braking gene network in *Drosophila* epithelia which tightly controls cell proliferation, apoptosis and cell competition *via* the Hpo pathway and dMyc expression. Importantly, YAP deregulation has been reported in several types of human cancers [54]–[56], therefore the mechanism of clonal expansion of Hpo pathway mutant cells in *Drosophila* might be relevant to understand tumor progression in mammals.

## Materials and Methods

### Genotypes and clonal analysis

The fly strains used in the present work were obtained by the Bloomington Stock Center and are described at http://flybase.bio.indiana.edu. The following strains were instead obtained by: *w*; *UAS*-*yki* (D Pan); *yw*, *tubFRTdmycFRTGal4* and *yw*, *dmyc* ^PL35^, *actFRTy^+^FRTGal4* (P Gallant); *w*, *hs*-*FLP*; *actFRTy* ^+^ *FRTGal4*, *UAS*-*GFP* (B Edgar); *w*; *FRT40A*, *ds^D36^* (I Rodríguez). The UAS-RNAi constructs for *dmyc*, *sd and hth* were obtained from the VDRC.

All experiments were carried out at 25°C unless otherwise indicated.

MARCM UAS-*yki* twin-spot clones were induced at different stages of development by a 35-minutes heat shock at 37°C and larvae of the following genotype were dissected at either 84-100h AEL or 120h AEL: *yw*, hs*-Flp*, *tub-Gal4*, UAS-*GFP*; FRT42D, *tub-Gal80*/FRT42D, Ubi-*GFP*; UAS-*yki*/+. Clones of the same genotype were induced 54–66 h AEL and dissected 48h after a 20-minutes heat shock (Figure S1). For FRT-Flp twin analysis, the following hypomorphic or null alleles were used: *ds^D36^*, *ft^G-rv^*, *ex^E1^*, *wts^X1^*, *yki^B5^*. Loss-of-function clones of *ds*, *ft*, *ex* and *wts* in either wild-type or mutant backgrounds overexpressing different transgenes in the posterior compartment were induced at 48–72h AEL by 1 hour heat shock at 37°C. Larvae of the following genotype were dissected at 120h AEL:

*yw*, hs-*Flp*; FRT40A, Ubi-*GFP*/FRT40A, *ds* ^D36^ or *ft* ^G-rv^ or *ex* ^E1^

*yw*, hs-*Flp*; FRT82B, Ubi-*GFP*/FRT82B, *wts* ^X1^

*yw*, hs-*Flp*; FRT40A, Ubi-*GFP*/FRT40A, *ds* ^D36^ or *ft* ^G-rv^ or *ex* ^E1^; *hh*-Gal4/UAS-*dmyc*

*yw*, hs-*Flp*; FRT40A, Ubi-*GFP*/FRT40A, *ft* ^G-rv^ or *ex* ^E1^; *hh*-Gal4/UAS-*dmyc*, UAS-*dIAP1*

The size of non-confluent clones was measured drawing each Z-stack of the confocal images using ImageJ software (http://rsbweb.nih.gov/ij). Afterwards the area of the clones was normalized dividing by the area of the wing pouch, considered as the territory encircled by the first outer folding of the wing. In Figure S1, the narrower window of clonal induction allowed us to compare clonal size without size normalization respect to the wing pouch. Statistical analysis was performed with Microsoft Excel and R (www.r-project.org). Statistical significance was determined by two tailed Student's t test and reported as the associated probability value (*P*).

Flp-Out clones were induced at 60h AEL by a 8-minutes heat shock at 37°C; imaginal discs of the following genotype were dissected at 120h AEL:

*yw*, hs-*Flp*; *act*FRT*y* ^+^FRT*Gal4*, UAS-*GFP*

*yw*, hs-*Flp*; UAS-*dmyc*RNAi/+; *act*FRT*y* ^+^FRT*Gal4*, UAS-*GFP*/+

*yw*, hs-*Flp*; *act*FRT*y* ^+^FRT*Gal4*, UAS-*GFP*/UAS-*yki*

*yw*, hs-*Flp*; UAS-*dmyc*RNAi/+; *act*FRT*y* ^+^FRT*Gal4*, UAS-*GFP*/UAS-*yki*.

*yw*, hs-*Flp*/*w*, *dmyc>lacZ* ^G0354^; *act*FRT*y* ^+^FRT*Gal4*, UAS-*GFP*/UAS-*yki*.

Cell competition assays were performed at 72h AEL inducing a 40-minutes heat shock at 36°C. Larvae of the following genotype were dissected at 120h AEL:

*yw*, *tub*FRT*y* ^+^FRT*Gal4*/hs-*Flp*; UAS-*GFP/+*

*yw*, *tub*FRT*y* ^+^FRT*Gal4*/hs-*Flp*; UAS-*GFP/+*; UAS-*yki/+*

*yw*, *tub*FRT*dmyc*FRT*Gal4/hs-Flp*; UAS-*GFP/+*; UAS-*yki/+*

*yw*, *dmyc* ^PL35^, hs-*Flp*, *tub*FRT*dmyc*FRT*Gal4/+-Y*; UAS-*GFP/+*; UAS-*yki/+*.

MARCM *yki* clones overexpressing p35, dMyc or both were generated at 48–72h AEL by a 45-minutes heat shock at 37°C and larvae were dissected 48h later.

### Immunofluorescence

Immunostainings were performed using standard protocols. The following primary antibodies were used: mouse anti-dMyc (1∶5, P Gallant), mouse anti-En (1∶50, DSHB), rabbit anti-active Caspase 3 (1∶100, Cell Signaling Technology), rabbit anti-p35 (1∶1000, Stratagene), rabbit anti-Ds (1∶100, D Strutt), rabbit anti-Hth (1∶400, A Salzberg, [57]), mouse anti-dIAP1 (1∶100, B Hay) and rabbit anti-ßGal (1∶400, F Graziani). Anti-mouse and anti-rabbit Alexa Fluor 555 (1∶200) (Molecular Probes) and anti-mouse Cy5 (1∶200) (Jackson Laboratories) against corresponding primary antibodies were used as secondary antibodies. Imaginal discs were mounted in Vectashield (Vector Laboratories) for confocal imaging. Single Z stacks were acquired with Leica SP2 and SP5 confocal microscopes. Images for Figure 4 and Figure 6 were captured with an epifluorescence Nikon 90i microscope. Entire images were elaborated with Photoshop CS2 (Adobe) and the projections along the Z axis were rebuilt starting from 35–55 Z stacks using the ImageJ public software (NIH). For measurements of dMyc abundance, fluorescence intensity was calculated using the ImageJ public software (NIH) as the average gray value within selectioned portions of confocal Z stacks. For measurement of active Caspase 3 signal outside UAS-*dmyc*-RNAi; UAS-*yki* and UAS-*yki* clones, staged wing discs were chosen containing as few clones as possible and single cells positive to active Caspase 3 observed at a maximum distance of five nuclei (counterstained with DAPI) from the border of the clone were counted on confocal Z stacks. *In situ* hybridization was performed with a full length *dmyc* probe [9] on wing imaginal discs of L3 larvae expressing UAS-*GFP*, UAS-*dmyc* or UAS-*yki* under the control of *dpp*-Gal4. RNA *in situ* hybridization was carried out using digoxigenin-labeled RNA probes [58].

### Luciferase transient expression assays

*Drosophila* S2 cells were grown at 25°C in Schneider medium (GIBCO) supplemented with 10% heat-inactivated FCS and 100 units of penicillin.

1189 base pairs located in the second intron of the *dmyc* sequence (Figure 3H) were subcloned into a pGL3-*firefly* vector (Promega) and co-transfected with Sd and/or Yki-expressing pAc5.1/V5-HisB plasmids [28] using Effectene Qiagen Transfection Kit. The primers used for that purpose were:

5′ CAGCGGTACCAGTTTGCTGTCCTCTGC 3′

5′GCACTCTAGAGCCATGCGGAATTGTGCG 3′.

The PCR product was first cloned in pCR 2.1 TOPO-TA (Sigma) and then subcloned in KpnI/XhoI sites of pGL3 Promoter vector. For luciferase transient expression assays, 2×10^4^ cells were plated in 96-well dishes. Cells were harvested at 48 hours after transfection and luciferase activity was measured using the Dual-Luciferase reporter assay system (Promega). Dual-Luciferase measurements were performed using a FLUOstar Optima luminometer (BMG Labtech) and normalized to the *Renilla* luciferase activity using pAct5C-seapansy as an internal control. All transient expression data reported in this paper represent the means from three parallel experiments, each performed in triplicate. Average relative luciferase activity was graphed and statistically analyzed by the Student's *t*-test.

## Supporting Information

Figure S1 *yki* ^over^ cells supercompetitive behavior is indeed visible at 48h after induction. (A,B) *yw*, hs*-Flp*, *tub-Gal4*, UAS-*GFP*; FRT42D, *tub-Gal80*/FRT42D, Ubi-*GFP* (A) and *yw*, hs-*Flp,tub-Gal4*, UAS-*GFP*; FRT42D, *tub-Gal80*/FRT42D, Ubi-*GFP*; UAS-*yki*/+ (B) clones induced at 54–66h AEL and dissected 48h after the heat-shock. Wild type and *yki* ^over^ clones are GFP^2+^ and twin clones are marked by the lack of GFP. Cell death is assayed by active Caspase 3 inmunoreactivity in red. Note that cell death is almost absent in the wild type experiment (A″) and marks wild type cells in the *yki* ^over^ experiment (B″). (C–F) Histograms showing the surface area of wild type and *yki* ^over^ clones and respective twins. (C,F) Wild type clones (C) and their twins (D) display the same size profile. (E) The size profile indicates that *yki* ^over^ clones are larger than wild type controls (C) as than their wild type twins (F) after only 48h of growth in the wing. SEM = Standard Error of the Mean. *P*<0.0001.
(1.60 MB TIF)

Figure S2 Hpo pathway LOFs induce cell competition. (A,B) Activated Caspase 3 staining of *yw*, hs-*Flp*/*+*; *ft* ^G-rv^, FRT40A/*Ubi>GFPnls*, FRT40A discs in which mutant clones (0xGFP) were grown for 48 hours (48–96 in A and 72–120 in B); apoptotic cell death occurs mainly in wild type cells surrounding the mutant clones (arrowheads). (C,D) Activated Caspase 3 staining of *yw*, hs-*Flp*/*+*; *ex* ^E1^, FRT40A/*Ubi>GFPnls*, FRT40A (C) and hs-*Flp*/*+*; *wts* ^X1^, FRT82B/*Ubi>GFPnls*, FRT82B (D) discs in which mutant clones (0xGFP) were grown for a longer period (48–108 and 48–120 hours respectively); apoptotic death is visible in both wild type (D, arrowheads) and mutant (D, arrows) cells.
(2.75 MB TIF)

Figure S3 dMyc upregulation in *yki* ^over^ clones is cell-autonomous. dMyc staining in *yw*, hs-*Flp*/+; *actFTRy+FRTGal4*, UAS-*GFP*/UAS-*yki* imaginal wing discs. *yki* ^over^ clones (GFP^+^, in green) express high levels of dMyc (in red) compared to the endogenous background. Z-section indicates that dMyc (in red) up-regulation is confined to *yki*-expressing cells (in green).
(0.51 MB TIF)

Figure S4 Hpo overexpression reduces dMyc protein levels. (A) dMyc staining in *w*; *sal>Gal4/+*; UAS-*p35*/+ imaginal wing discs. (B–C) dMyc staining of late (B) and early (C) *w*; *sal>Gal4/+*; UAS-*Hpo/+*; UAS-*p35/+* imaginal wing discs. p35 is shown in the green channel and dMyc in red. As can be observed in the Z-sections dMyc abundance is lower inside the *sal* domain. The position of Z-section is indicated by white bars in the surface view of the wing discs.
(2.76 MB TIF)

Figure S5 *dmyc* is transcriptionally upregulated in *ft* mutant clones. ßGal staining (red) of *dmyc>lacZ* ^G0354^/hs-*Flp*; *ft* ^G-rv^, FRT40/*UbiGFPnls*, imaginal wing discs. As can be observed, a robust activation of *dmyc* regulatory sequences is visible within the mutant clones (arrows). Larvae were dissected at 120h AEL.
(0.93 MB TIF)

Figure S6 Hth is necessary for Yki-induced dMyc overexpression in the presumptive thoracic region of the wing disc. (A) Wing from a *w*; UAS-*sd*-RNAi/*en>Gal4* individual. For UAS-*sd*-RNAi line validation, we induced the expression of the *sd*-RNAi construct in the posterior compartment of the wing by means of the *engrailed* (*en*) promoter. As can be observed, the wing lacks the posterior compartment (green-colored in the insert). (B) dMyc staining in *yw*, hs-*Flp/+*; UAS-*hth*-RNAi/*+*; UAS-*yki/actFTRy+FRTGal4*, UAS-*GFP* imaginal wing discs. Note that mutant clones (GFP^+^) overgrow and overexpress dMyc in the wing pouch region (white arrowhead) and not in the notum region (yellow arrows). (C) For UAS-*hth*-RNAi line validation, we stained for Hth [57] *yw*, hs-*Flp*/+; UAS-*hth*-RNAi/+; *actFTRy+FRTGal4*, UAS-*GFP*/+ wing discs. Hth expression is lacking in the clone originated in the pleural region (arrow).
(1.49 MB TIF)

Figure S7 *dmyc* is involved in the competitive ability of *yki*. (A) dMyc levels are strongly affected inside *dmyc*-RNAi; *yki* ^over^ clones. (A′) A projection along the Z axis of the clone presented in Figure A is shown. The percentage of dMyc abundance reduction inside the mutant clones calculated as the abatement of fluorescence intensity (see Methods - Immunofluorescence) with respect to the neighboring tissue was 62% on average (n = 12). (B) *dmyc*-RNAi; *yki^over^* clones display a reduced non-autonomous apoptotic activity (yellow arrows, see Methods - Immunofluorescence - for calculation) compared to *yki^over^* clones (see Figure 1). (C) *yki* ^over^ clones can compete in a high *dmyc* level background, where wild type clones fail to grow; clones were induced at 66–78h AEL and allowed to grow until 120h AEL. In red, staining for dMyc indicates that dMyc levels are quite similar inside the *yki* ^over^ clone and in the *tub>dmyc* background. (C′) A projection along the Z axis of the clone presented in figure C is shown. (D–E) In adult wings, *tub>yki* ^over^ clones generated in a wild type background (D) are bigger than *tub>yki* ^over^ clones generated in a *tub>dmyc* background (red arrows, E) confirming the results illustrated in Figure 6. Clones were induced at 66–78h AEL and survived up to the adult stage.
(1.18 MB TIF)

Figure S8 dMyc overexpression boosts proliferation in *ds* mutant cells. (A) *ds* LOF clones (0xGFP) generated in a background where posterior cells ectopically express *dmyc* under the control of the *hh* promoter (on the right). dMyc overexpression strongly enhances the proliferative activity of *ds* mutant cells; mutant clones are larger in dMyc-expressing territories (posterior compartment in C) than in a wild type background (anterior compartment in B). SEM = Standard Error of the Mean. *P*<0.001.
(0.67 MB TIF)

Figure S9 dMyc overexpression boosts proliferation of Hpo pathway mutant cells also when wild type cells are protected from cell death. *ft* (A–C) and *ex* (D–F) LOF clones (0xGFP) generated in a background where posterior (P) cells ectopically coexpress *dmyc* and *dIAP1* under the control of *hh-Gal4* (A and P compartments are separated by a white line in A and D; P is on the right). dMyc overexpression enhances the proliferative activity of *ft* (A–C) and *ex* (D–F) mutant cells; mutant clones are larger in dMyc-dIAP1 expressing territories (P compartment in histograms C and F) than in a wild type background (A compartment in histograms B and E). All panels show Caspase 3 staining in red and dIAP1 in blue. SEM = Standard Error of the Mean. *P*<0.001.
(2.59 MB TIF)

Figure S10 *dmyc* fails to rescue *yki* LOF upon inhibition of cell death. Three types of *yki* LOF clones were induced through the MARCM system. In (A), *yki* mutant clones were generated while overexpressing the antiapoptotic protein p35 (in red). (B) Overexpression of *dmyc* fails to rescue *yki* mutant cells viability and Caspase 3 activation (red arrows). (C) The overexpression of p35 and *dmyc* together also fails to rescue *yki* mutant cells viability.
(1.47 MB TIF)

## References

[1] Morata G, Ripoll P (1975). Minutes: mutants of Drosophila autonomously affecting cell division rate.. Dev Biol.

[2] Moreno E (2008). Is cell competition relevant to cancer?. Nat Rev Cancer.

[3] Martín FA, Herrera FC, Morata G (2009). Cell competition, growth and size control in the Drosophila wing imaginal disc.. Development.

[4] de la Cova C, Abril M, Bellosta P, Gallant P, Johnston LA (2004). Drosophila myc regulates organ size by inducing cell competition.. Cell.

[5] Moreno E, Basler K (2004). dMyc transforms cells into super-competitors.. Cell.

[6] Baker NE (2008). Cell competition and its possible relation to cancer.. Cancer Res.

[7] Froldi F, Ziosi M, Garoia F, Pession A, Grzeschik NA (2010). The lethal giant larvae tumour suppressor mutation requires dMyc oncoprotein to promote clonal malignancy.. BMC Biol.

[8] Oster SK, Ho CS, Soucie EL, Penn LZ (2002). The myc oncogene: Marvelous Complex.. Adv Cancer Res.

[9] Johnston LA, Prober DA, Edgar BA, Eisenman RN, Gallant P (1999). Drosophila myc regulates cellular growth during development.. Cell.

[10] Meyer N, Kim SS, Penn LZ (2006). The Oscar-worthy role of Myc in apoptosis.. Semin Cancer Biol.

[11] Montero L, Müller N, Gallant P (2008). Induction of Apoptosis by Drosophila Myc.. Genesis.

[12] Grewal SS, Li L, Orian A, Eisenman RN, Edgar BA (2005). Myc-dependent regulation of ribosomal RNA synthesis during Drosophila development.. Nat Cell Biol.

[13] Vita M, Henriksson M (2006). The Myc oncoprotein as a therapeutic target for human cancer.. Semin Cancer Biol.

[14] Land H, Parada LF, Weinberg RA (1983). Cellular oncogenes and multistep carcinogenesis.. Science.

[15] Zhan L, Rosenberg A, Bergami KC, Yu M, Xuan Z (2008). Deregulation of Scribble Promotes Mammary Tumorigenesis and Reveals a Role for Cell Polarity in Carcinoma.. Cell.

[16] Huang J, Wu S, Barrera J, Matthews K, Pan D (2005). The Hippo signaling pathway coordinately regulates cell proliferation and apoptosis by inactivating Yorkie, the Drosophila homolog of YAP.. Cell.

[17] Dong J, Feldmann G, Huang J, Wu S, Zhang N (2007). Elucidation of a universal size-control mechanism in Drosophila and mammals.. Cell.

[18] Saucedo LJ, Edgar B (2007). Filling out the Hippo pathway.. Nat Rev Mol Cell Biol.

[19] Tyler DM, Li W, Zhuo N, Pellock B, Baker NE (2006). Genes affecting cell competition in Drosophila.. Genetics.

[20] Harvey K, Tapon N (2007). The Salvador-Warts-Hippo pathway - an emerging tumour-suppressor network.. Nat Rev Cancer.

[21] Oh H, Irvine KD (2009). In vivo analysis of Yorkie phosphorilation sites.. Oncogene.

[22] Ren F, Zhang L, Jiang J (2009). Hippo signaling regulates Yorkie nuclear localization and activity through 14-3-3 dependent and independent mechanisms.. Dev Biol.

[23] Nicolay BN, Frolov MV (2008). Context-dependent requirement for dE2F during oncogenic proliferation.. PLoS Genet.

[24] Nolo R, Morrison CM, Tao C, Zhang X, Halder G (2006). The bantam MicroRNA is a target of the Hippo tumor-suppressor pathway.. Curr Biol.

[25] Thompson BJ, Cohen SM (2006). The Hippo pathway regulates the bantam microRNA to control cell proliferation and apoptosis in Drosophila.. Cell.

[26] Baena-Lopez LA, Rodriguez I, Baonza A (2008). The tumor suppressor genes dachsous and fat modulate different signalling pathways by regulating dally and dally-like.. Proc Natl Acad Sci USA.

[27] Goulev Y, Fauny JD, Gonzalez-Marti B, Flagiello D, Silber J (2008). SCALLOPED Interacts with YORKIE, the Nuclear Effector of the Hippo Tumor-Suppressor Pathway in Drosophila.. Curr Biol.

[28] Wu S, Liu Y, Zheng Q, Dong J, Pan D (2008). The TEAD/TEF family protein Scalopped mediates transcriptional output of the Hippo growth-regulatory pathway.. Dev Cell.

[29] Zhang L, Ren F, Zhang Q, Chen Y, Wang B (2008). The TEAD/TEF family of transcription factor Scalopped mediates Hippo signaling in organ size control.. Dev Cell.

[30] Zhao B, Ye X, Yu J, Li L, Li W (2008). TEAD mediates YAP-dependent gene induction and growth control.. Genes Dev.

[31] Peng HW, Slattery M, Mann RS (2009). Transcription factor choice in the Hippo signaling pathway: homothorax and yorkie regulation of the microRNA bantam, in the progenitor domain of the Drosophila eye imaginal disc.. Genes Dev.

[32] Willecke M, Hamaratoglu F, Kango-Singh M, Udan R, Chen C (2006). The Fat Cadherin Acts through the Hippo Tumor-Suppressor Pathway to Regulate Tissue Size.. Curr Biol.

[33] Silva E, Tsatskis Y, Gardano L, Tapon N, McNeill H (2006). The Tumor-Suppressor Gene fat Controls Tissue Growth Upstream of Expanded in the Hippo Signaling Pathway.. Curr Biol.

[34] Cho E, Feng Y, Rauskolb C, Maitra S, Fehon R (2006). Delineation of a Fat tumor suppressor pathway.. Nat Genet.

[35] Bennett FC, Harvey KF (2006). Fat Cadherin Modulates Organ Size in Drosophila via the Salvador/Warts/Hippo Signaling Pathway.. Curr Biol.

[36] Cho E, Irvine KD (2004). Action of fat, four-jointed, dachsous and dachs in distal-to-proximal wing signaling.. Development.

[37] Feng Y, Irvine KD (2007). Fat and expanded act in parallel to regulate growth through warts.. Proc Natl Acad Sci USA.

[38] Willecke M, Hamaratoglu F, Sansores-Garcia L, Tao C, Halder G (2008). Boundaries of Dachsous Cadherin activity modulate the Hippo signaling pathway to induce cell proliferation.. Proc Natl Acad Sci USA.

[39] Hamaratoglu F, Willecke M, Kango-Singh M, Nolo R, Hyun E (2006). The tumour suppressor genes NF2/Merlin and Expanded act through Hippo signalling to regulate cell proliferation and apoptosis.. Nat Cell Biol.

[40] Wu S, Huang J, Dong J, Pan D (2003). hippo encodes a Ste-20 family protein kinase that restricts cell proliferation and promotes apoptosis in conjunction with salvador and warts.. Cell.

[41] Reddy BVVG, Irvine KD (2008). The Fat and warts signaling pathways: new insights into their regulation, mechanism and conservation.. Development.

[42] Moreno E, Basler K, Morata G (2002). Cells compete for decapentaplegic survival factor to prevent apoptosis in Drosophila wing development.. Nature.

[43] Li W, Baker NE (2007). Engulfment is required for cell competition.. Cell.

[44] Senoo-Matsuda N, Johnston LA (2007). Soluble factors mediate competitive and cooperative interactions between cells expressing different levels of Drosophila Myc.. Proc Natl Acad Sci USA.

[45] Garoia F, Grifoni D, Trotta V, Guerra D, Pezzoli MC (2005). The tumor suppressor gene fat modulates the EGFR-mediated proliferation control in the imaginal tissues of Drosophila melanogaster.. Mech Dev.

[46] Peter A, Schöttler P, Werner M, Beinert N, Dowe G (2002). Mapping and identification of essential gene functions on the X chromosome of Drosophila.. EMBO Rep.

[47] Cranna N, Quinn L (2009). Impact of steroid hormone signals on Drosophila cell cycle during development.. Cell Div.

[48] Xiao JH, Davidson I, Matthes H, Garnier JM, Chambon P (1991). Cloning, expression, and transcriptional properties of the human enhancer factor TEF-1.. Cell.

[49] Larkin SB, Farrance IK, Ordahl CP (1996). Flanking sequences modulate the cell specificity of M-CAT elements.. Mol Cell Biol.

[50] Bourbon HM, Gonzy-Treboul G, Peronnet F, Alin MF, Ardourel C (2002). A P-insertion screen identifying novel X-linked essential genes in Drosophila.. Mech Dev.

[51] Benassayag C, Montero L, Colombie N, Gallant P, Cribbs D (2005). Human c-Myc isoforms differentially regulate cell growth and apoptosis in Drosophila melanogaster.. Mol Cell Biol.

[52] Martín FA, Peréz-Garijo A, Morata G (2009). Apoptosis in Drosophila: compensatory proliferation and undead cells.. Int J Dev Biol.

[53] Oh H, Irvine KD (2008). In vivo regulation of Yorkie phosphorylation and localization.. Development.

[54] Lam-Himlin DM, Daniels JA, Gayyed MF, Dong J, Maitra A (2006). The hippo pathway in human upper gastrointestinal dysplasia and carcinoma: a novel oncogenic pathway.. Int J Gastrointest Cancer.

[55] Steinhardt AA, Gayyed MF, Klein AP, Dong J, Maitra A (2008). Expression of Yes-associated protein in common solid tumors.. Hum Pathol.

[56] Overholtzer M, Zhang J, Smolen GA, Muir B, Li W (2006). Transforming properties of YAP, a candidate oncogene on the chromosome 11q22 amplicon.. Proc Natl Acad Sci USA.

[57] Kurant E, Pai CY, Sharf R, Halachmi N, Sun YH (1998). Dorsotonals/homothorax, the Drosophila homologue of meis1, interacts with extradenticle in patterning of the embryonic PNS.. Development.

[58] Johnston LA, Edgar BA (1998). Wingless and Notch regulate cell-cycle arrest in the developing Drosophila wing.. Nature.

[59] De la Cova C, Johnston LA (2006). Myc in model organisms: a view from the fly room.. Sem Cancer Biol.

