# Supplementary figures and images for: dMyc Functions Downstream of Yorkie to Promote the Supercompetitive Behavior of Hippo Pathway Mutant Cells
Source: PLoS Genet. 2010 Sep 23;6(9):e1001140. doi: 10.1371/journal.pgen.1001140 (PMC2944792; doi:10.1371/journal.pgen.1001140)

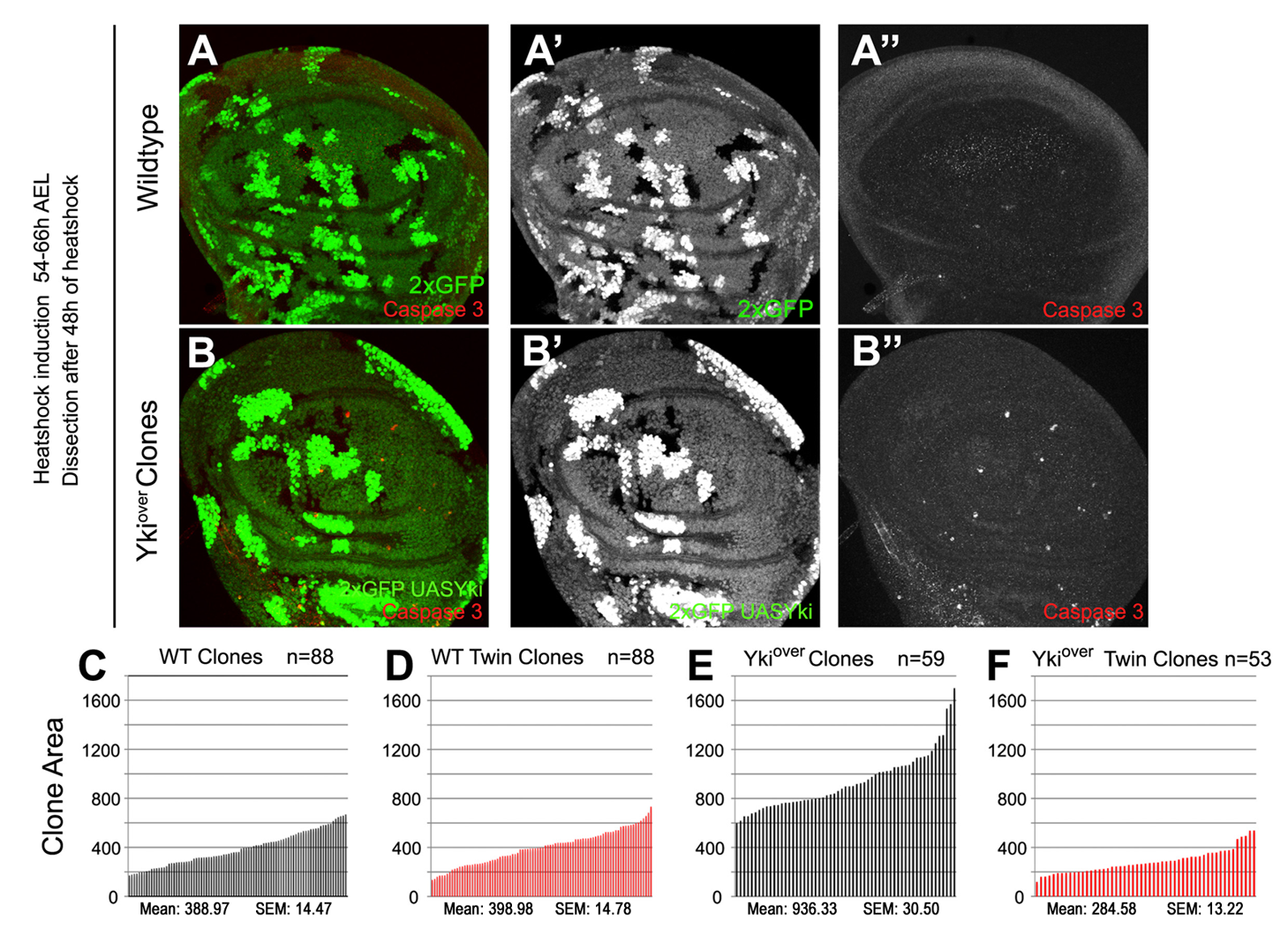

Supplement: Figure S1 — yki over cells supercompetitive behavior is indeed visible at 48h after induction. (A,B) yw, hs-Flp, tub-Gal4, UAS-GFP; FRT42D, tub-Gal80/FRT42D, Ubi-GFP (A) and yw, hs-Flp,tub-Gal4, UAS-GFP; FRT42D, tub-Gal80/FRT42D, Ubi-GFP; UAS-yki/+ (B) clones induced at 54–66h AEL and dissected 48h after the heat-shock. Wild type and yki over clones are GFP2+ and twin clones are marked by the lack of GFP. Cell death is assayed by active Caspase 3 inmunoreactivity in red. Note that cell death is almost absent in the wild type experiment (A″) and marks wild type cells in the yki over experiment (B″). (C–F) Histograms showing the surface area of wild type and yki over clones and respective twins. (C,F) Wild type clones (C) and their twins (D) display the same size profile. (E) The size profile indicates that yki over clones are larger than wild type controls (C) as than their wild type twins (F) after only 48h of growth in the wing. SEM = Standard Error of the Mean. P<0.0001. (1.60 MB TIF) [file pgen.1001140.s001.tif]

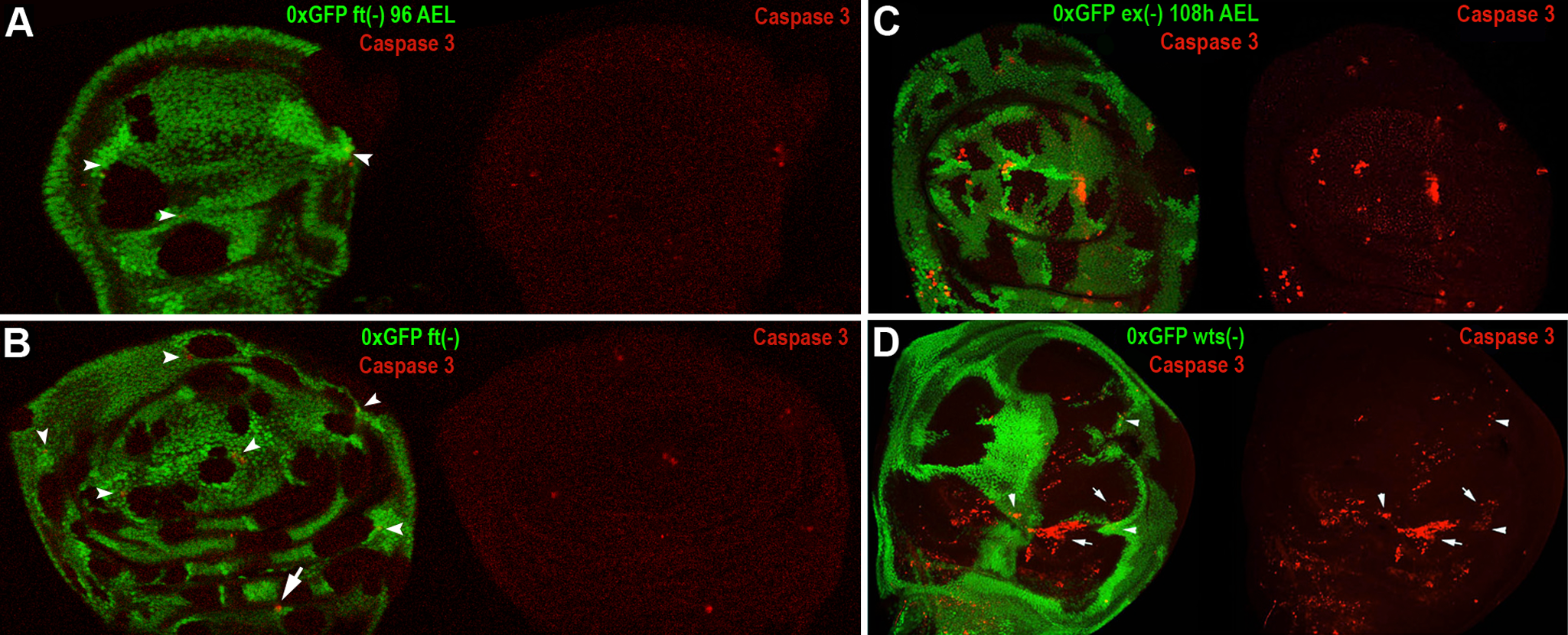

Supplement: Figure S2 — Hpo pathway LOFs induce cell competition. (A,B) Activated Caspase 3 staining of yw, hs-Flp/+; ft G-rv, FRT40A/Ubi>GFPnls, FRT40A discs in which mutant clones (0xGFP) were grown for 48 hours (48–96 in A and 72–120 in B); apoptotic cell death occurs mainly in wild type cells surrounding the mutant clones (arrowheads). (C,D) Activated Caspase 3 staining of yw, hs-Flp/+; ex E1, FRT40A/Ubi>GFPnls, FRT40A (C) and hs-Flp/+; wts X1, FRT82B/Ubi>GFPnls, FRT82B (D) discs in which mutant clones (0xGFP) were grown for a longer period (48–108 and 48–120 hours respectively); apoptotic death is visible in both wild type (D, arrowheads) and mutant (D, arrows) cells. (2.75 MB TIF) [file pgen.1001140.s002.tif]

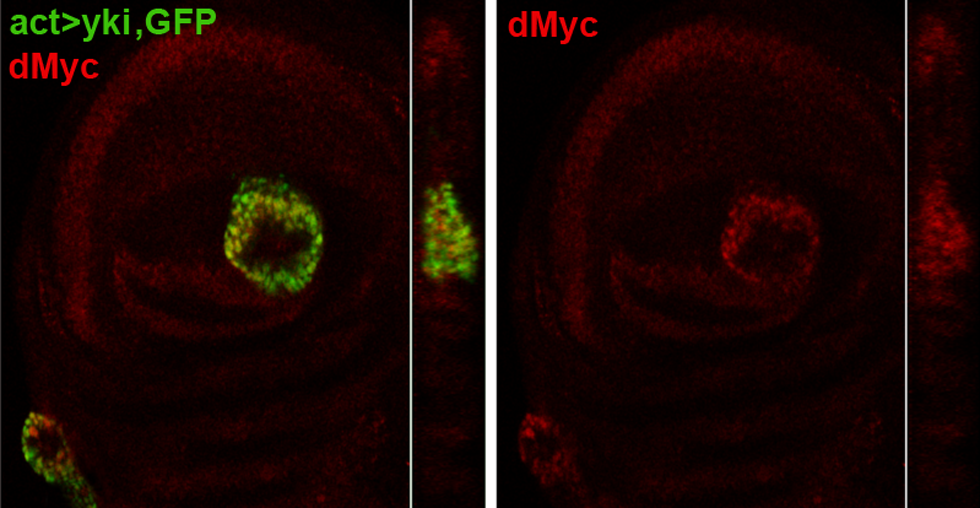

Supplement: Figure S3 — dMyc upregulation in yki over clones is cell-autonomous. dMyc staining in yw, hs-Flp/+; actFTRy+FRTGal4, UAS-GFP/UAS-yki imaginal wing discs. yki over clones (GFP+, in green) express high levels of dMyc (in red) compared to the endogenous background. Z-section indicates that dMyc (in red) up-regulation is confined to yki-expressing cells (in green). (0.51 MB TIF) [file pgen.1001140.s003.tif]

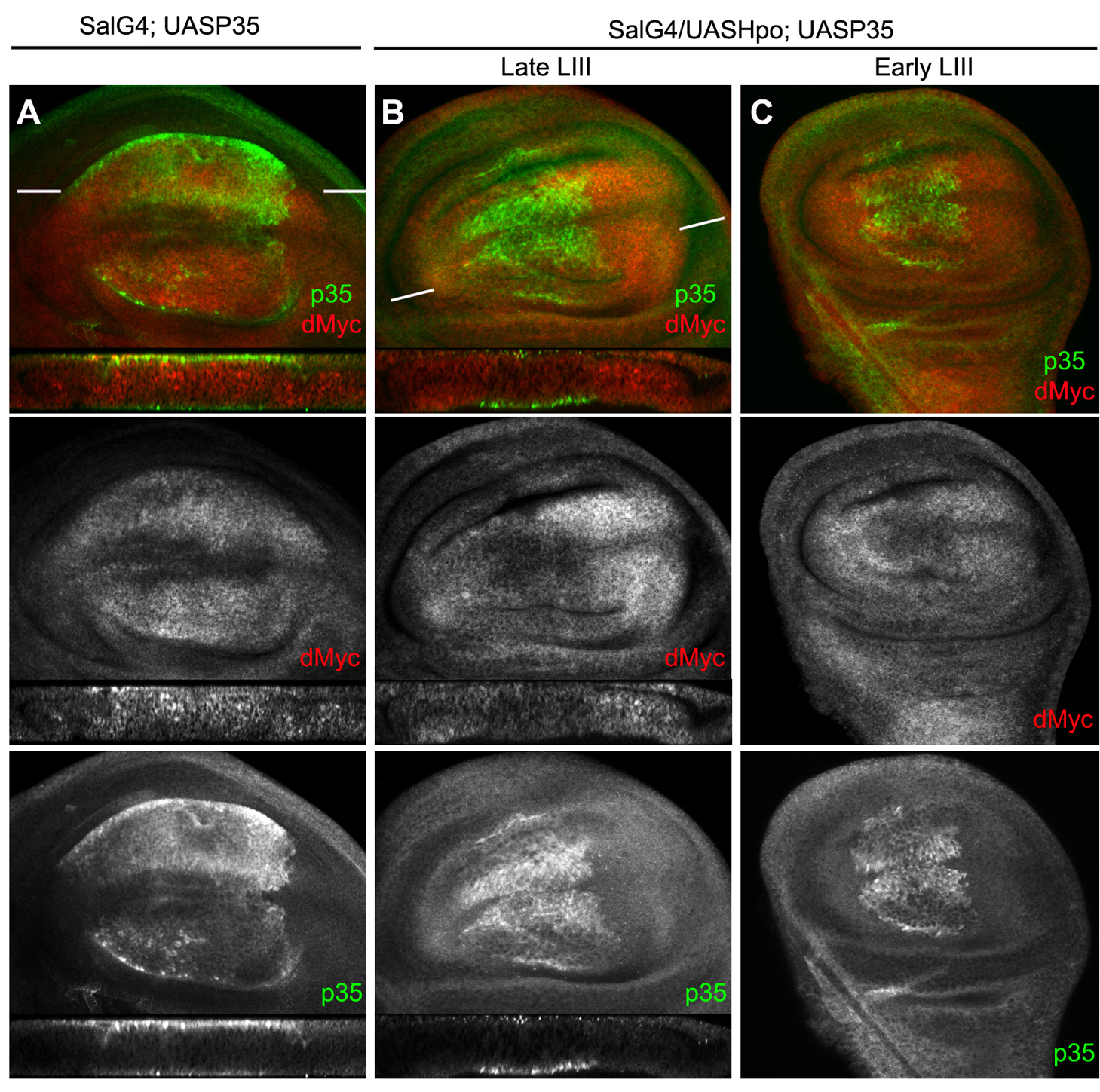

Supplement: Figure S4 — Hpo overexpression reduces dMyc protein levels. (A) dMyc staining in w; sal>Gal4/+; UAS-p35/+ imaginal wing discs. (B–C) dMyc staining of late (B) and early (C) w; sal>Gal4/+; UAS-Hpo/+; UAS-p35/+ imaginal wing discs. p35 is shown in the green channel and dMyc in red. As can be observed in the Z-sections dMyc abundance is lower inside the sal domain. The position of Z-section is indicated by white bars in the surface view of the wing discs. (2.76 MB TIF) [file pgen.1001140.s004.tif]

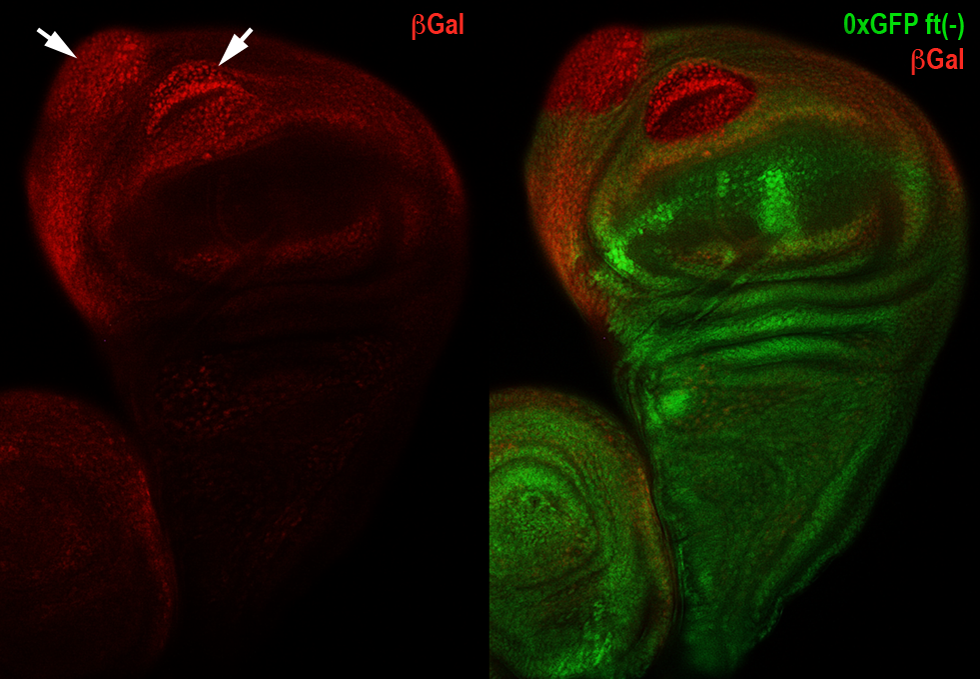

Supplement: Figure S5 — dmyc is transcriptionally upregulated in ft mutant clones. ßGal staining (red) of dmyc>lacZ G0354/hs-Flp; ft G-rv, FRT40/UbiGFPnls, imaginal wing discs. As can be observed, a robust activation of dmyc regulatory sequences is visible within the mutant clones (arrows). Larvae were dissected at 120h AEL. (0.93 MB TIF) [file pgen.1001140.s005.tif]

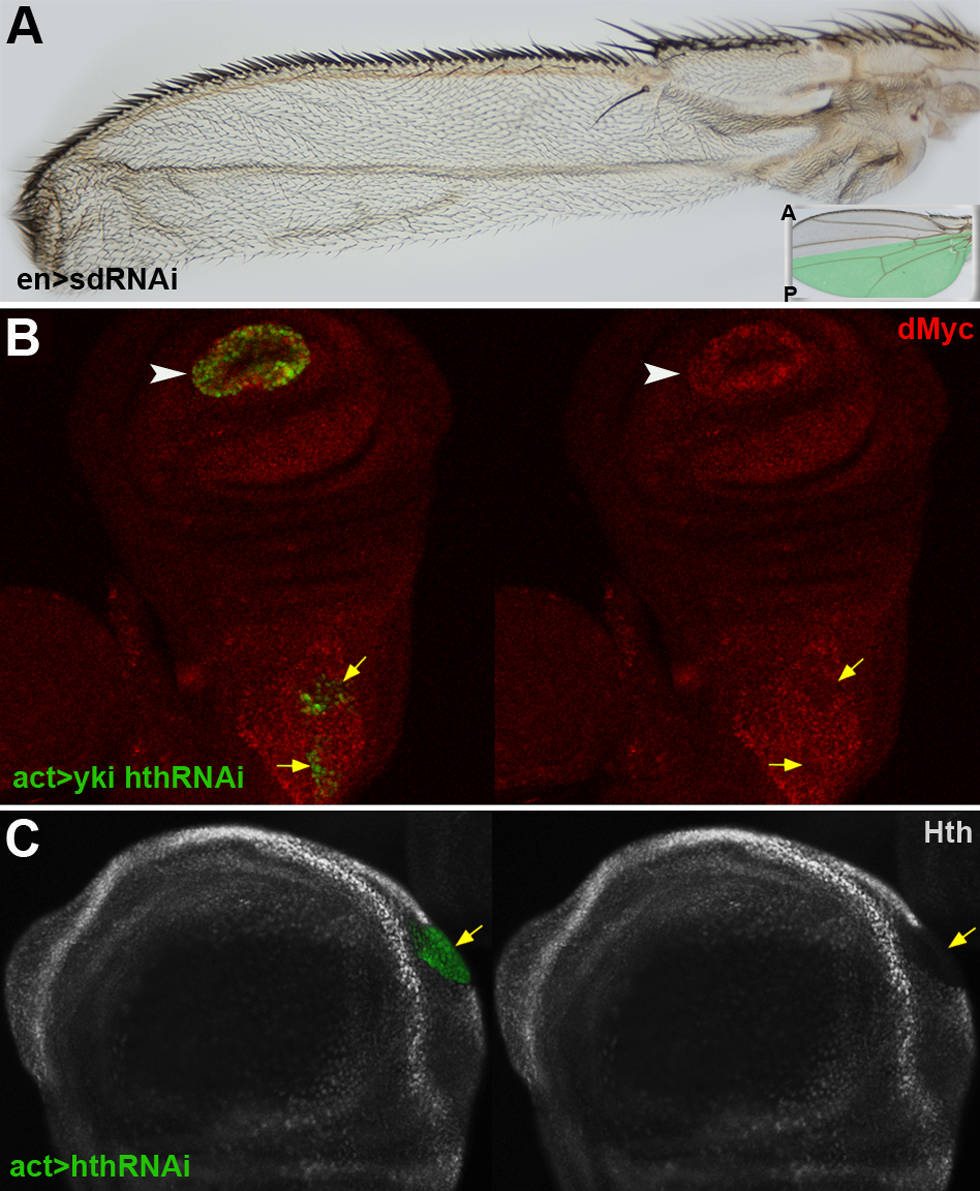

Supplement: Figure S6 — Hth is necessary for Yki-induced dMyc overexpression in the presumptive thoracic region of the wing disc. (A) Wing from a w; UAS-sd-RNAi/en>Gal4 individual. For UAS-sd-RNAi line validation, we induced the expression of the sd-RNAi construct in the posterior compartment of the wing by means of the engrailed (en) promoter. As can be observed, the wing lacks the posterior compartment (green-colored in the insert). (B) dMyc staining in yw, hs-Flp/+; UAS-hth-RNAi/+; UAS-yki/actFTRy+FRTGal4, UAS-GFP imaginal wing discs. Note that mutant clones (GFP+) overgrow and overexpress dMyc in the wing pouch region (white arrowhead) and not in the notum region (yellow arrows). (C) For UAS-hth-RNAi line validation, we stained for Hth [57] yw, hs-Flp/+; UAS-hth-RNAi/+; actFTRy+FRTGal4, UAS-GFP/+ wing discs. Hth expression is lacking in the clone originated in the pleural region (arrow). (1.49 MB TIF) [file pgen.1001140.s006.tif]

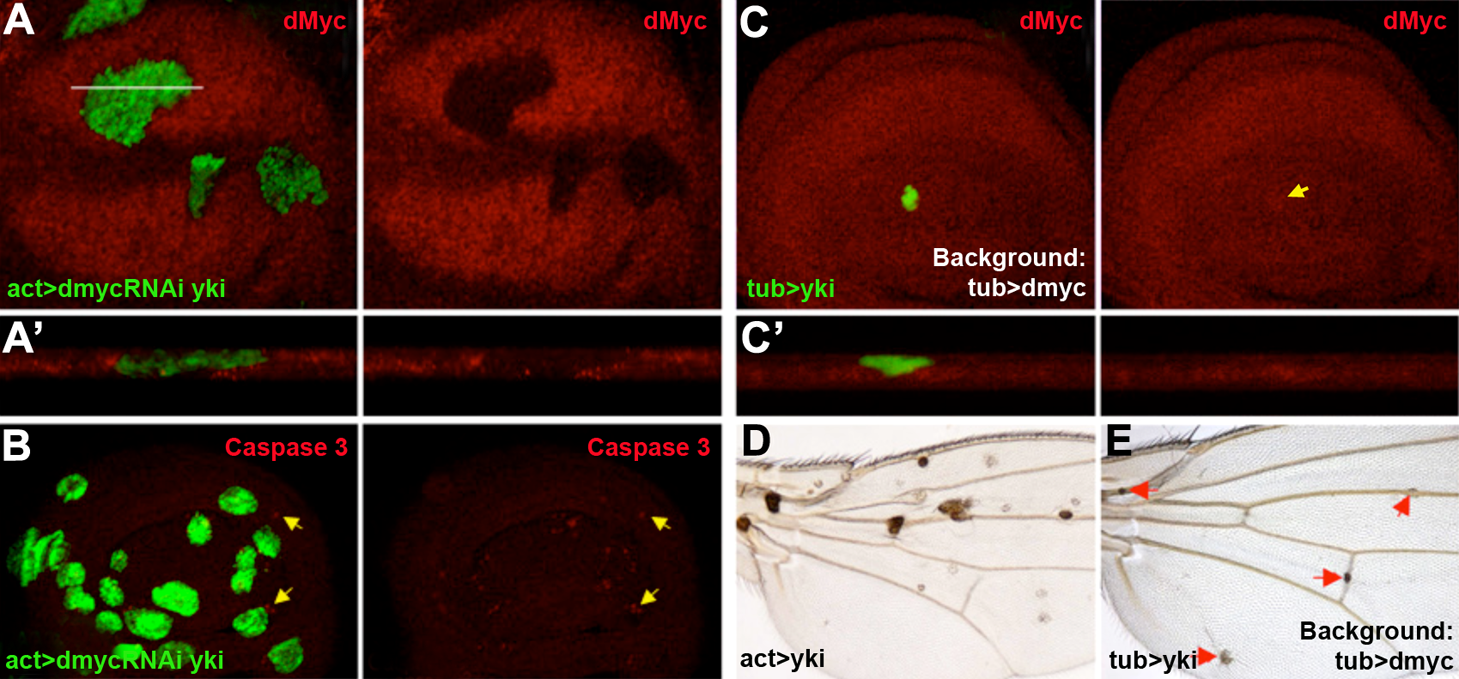

Supplement: Figure S7 — dmyc is involved in the competitive ability of yki. (A) dMyc levels are strongly affected inside dmyc-RNAi; yki over clones. (A′) A projection along the Z axis of the clone presented in Figure A is shown. The percentage of dMyc abundance reduction inside the mutant clones calculated as the abatement of fluorescence intensity (see Methods - Immunofluorescence) with respect to the neighboring tissue was 62% on average (n = 12). (B) dmyc-RNAi; ykiover clones display a reduced non-autonomous apoptotic activity (yellow arrows, see Methods - Immunofluorescence - for calculation) compared to ykiover clones (see Figure 1). (C) yki over clones can compete in a high dmyc level background, where wild type clones fail to grow; clones were induced at 66–78h AEL and allowed to grow until 120h AEL. In red, staining for dMyc indicates that dMyc levels are quite similar inside the yki over clone and in the tub>dmyc background. (C′) A projection along the Z axis of the clone presented in figure C is shown. (D–E) In adult wings, tub>yki over clones generated in a wild type background (D) are bigger than tub>yki over clones generated in a tub>dmyc background (red arrows, E) confirming the results illustrated in Figure 6. Clones were induced at 66–78h AEL and survived up to the adult stage. (1.18 MB TIF) [file pgen.1001140.s007.tif]

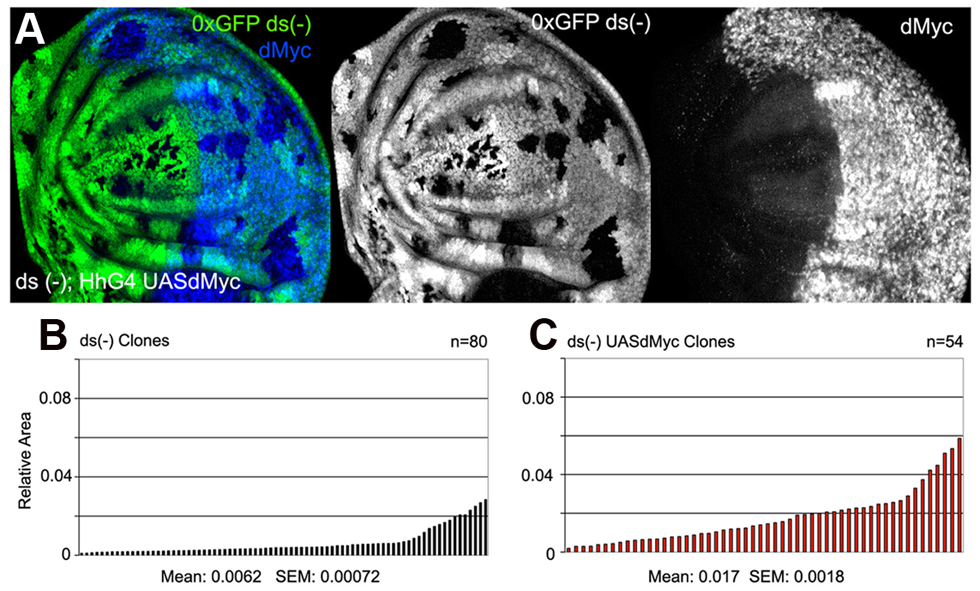

Supplement: Figure S8 — dMyc overexpression boosts proliferation in ds mutant cells. (A) ds LOF clones (0xGFP) generated in a background where posterior cells ectopically express dmyc under the control of the hh promoter (on the right). dMyc overexpression strongly enhances the proliferative activity of ds mutant cells; mutant clones are larger in dMyc-expressing territories (posterior compartment in C) than in a wild type background (anterior compartment in B). SEM = Standard Error of the Mean. P<0.001. (0.67 MB TIF) [file pgen.1001140.s008.tif]

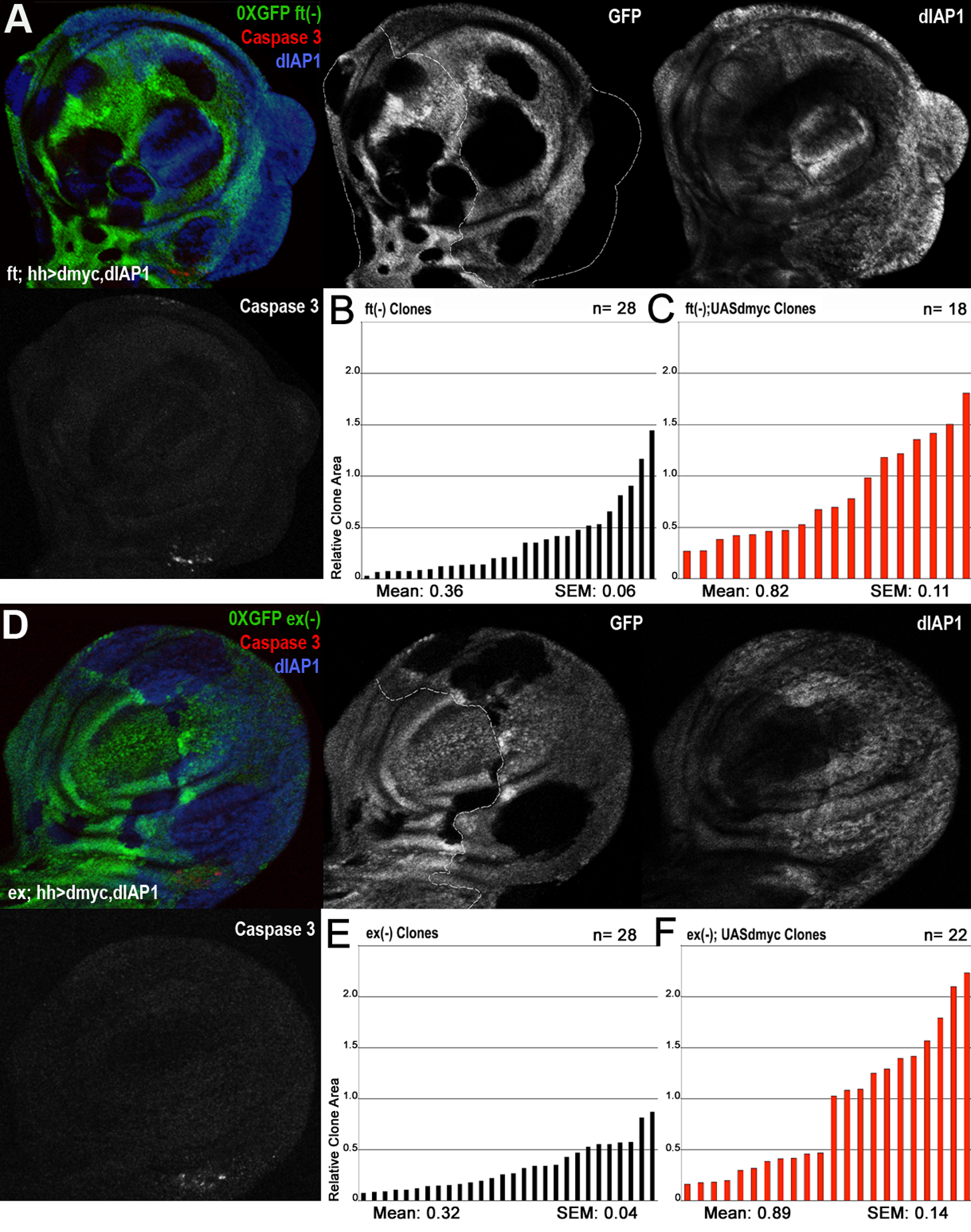

Supplement: Figure S9 — dMyc overexpression boosts proliferation of Hpo pathway mutant cells also when wild type cells are protected from cell death. ft (A–C) and ex (D–F) LOF clones (0xGFP) generated in a background where posterior (P) cells ectopically coexpress dmyc and dIAP1 under the control of hh-Gal4 (A and P compartments are separated by a white line in A and D; P is on the right). dMyc overexpression enhances the proliferative activity of ft (A–C) and ex (D–F) mutant cells; mutant clones are larger in dMyc-dIAP1 expressing territories (P compartment in histograms C and F) than in a wild type background (A compartment in histograms B and E). All panels show Caspase 3 staining in red and dIAP1 in blue. SEM = Standard Error of the Mean. P<0.001. (2.59 MB TIF) [file pgen.1001140.s009.tif]

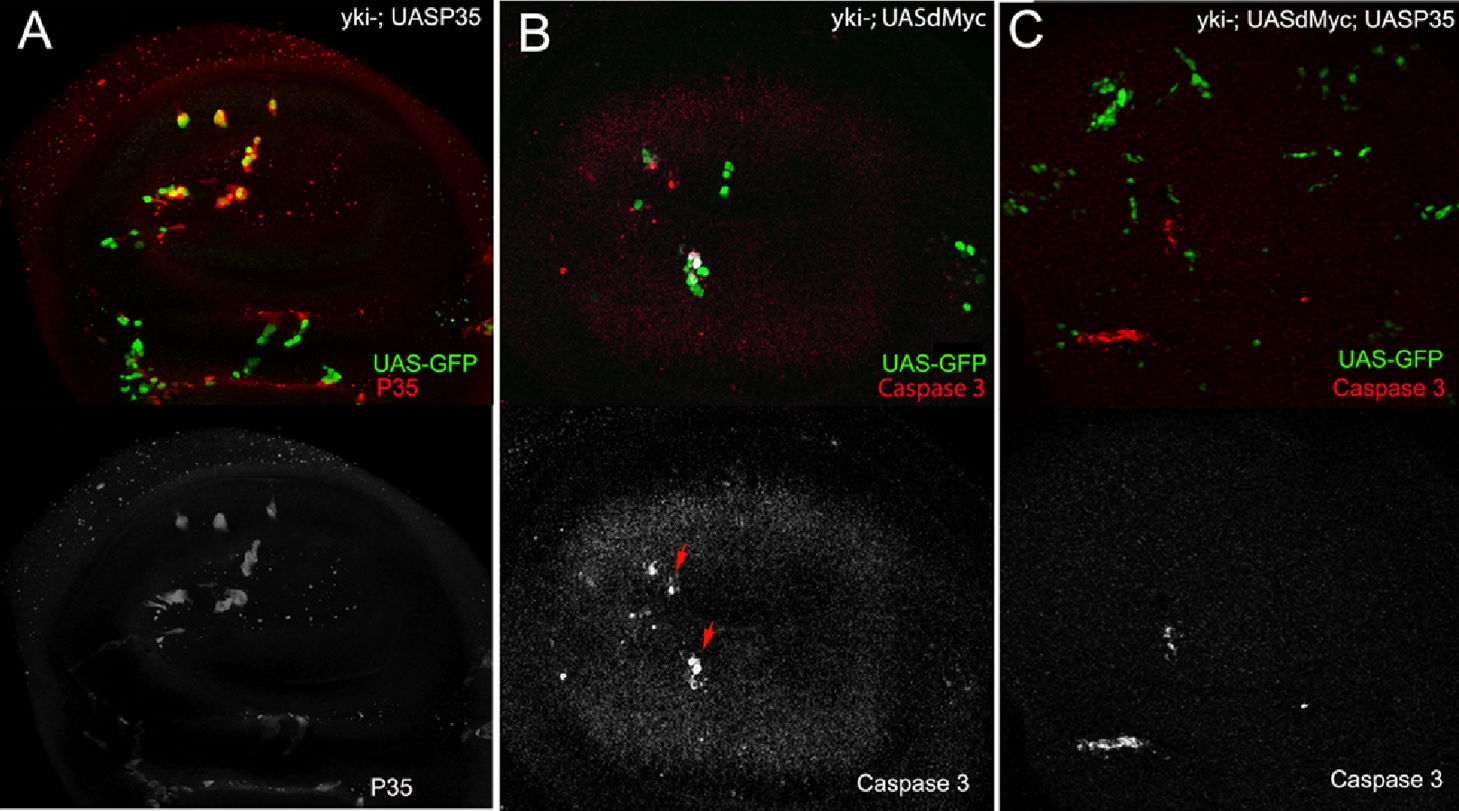

Supplement: Figure S10 — dmyc fails to rescue yki LOF upon inhibition of cell death. Three types of yki LOF clones were induced through the MARCM system. In (A), yki mutant clones were generated while overexpressing the antiapoptotic protein p35 (in red). (B) Overexpression of dmyc fails to rescue yki mutant cells viability and Caspase 3 activation (red arrows). (C) The overexpression of p35 and dmyc together also fails to rescue yki mutant cells viability. (1.47 MB TIF) [file pgen.1001140.s010.tif]
